# Primary Radiation Damage in a Strain-Engineering-Based SiGe/Si Heterostructure: A Molecular Dynamics Simulation

**DOI:** 10.3390/nano16030193

**Published:** 2026-01-30

**Authors:** Tian Xing, Shuhuan Liu, Qian Wang, Chao Wang, Yuchen Wang, Mathew Adefusika Adekoya, Xuan Wang, Xinkun Li, Huawei Sheng, Luyang Cai, Jiatong Tan, Yalei Yi, Zhongliang Li

**Affiliations:** 1 School of Nuclear Science and Technology, Xi’an Jiaotong University, Xi’an 710049, China; 2 National Key Laboratory for Metrology and Calibration Techniques, Beijing 102413, China; 3 Shaanxi Qinzhou Nuclear and Radiation Safety Technology Co., Ltd., Xi’an 710054, China

**Keywords:** molecular dynamics simulation, SiGe/Si heterostructure, collision cascade, overlapping effect, point defect, lattice temperature

## Abstract

Space-borne SiGe-based electronics are confronted with high-energy particles and may suffer from displacement damage effects. Here, primary radiation damage of a strain-engineering-based SiGe/Si heterostructure was investigated by molecular dynamics simulations in two cases of independent and overlapping collision cascades. The results showed that among 1 keV, 3 keV, and 5 keV primary knock-on atoms (PKAs) of Si and Ge, 3 keV Ge PKAs generated the most point defects at the heterointerface, which was associated with adequate PKA energy dissipated around the heterointerface. Meanwhile, the Frenkel pairs at the heterointerface continued increasing merely in the first three cascades and tended to annihilate subsequently, whereas the antisites both in the whole heterostructure and at the heterointerface accrued from the first to the fifth cascades. In addition, the spatial distribution of point defects surviving in each collision cascade was dominated by the melting region, and it could be superimposed on the subsequent ones during the overlapping cascades. Overall, this work explored the evolution of the defect and temperature as well as the overlapping effects during the collision cascades in a strain-engineering-based SiGe/Si heterostructure, which shall shed light on radiation effects of SiGe/Si heterostructures and pertinent radiation-hardening techniques of SiGe-based electronics.

## 1. Introduction

Space radiation environments may pose a severe threat to on-board electronic devices and bring about catastrophic failure of aerospace missions, which has driven researchers to delve into radiation damage of electronic devices and develop radiation-hardened and radiation-tolerant electronics, ensuring the robustness and reliability of electronic systems for space exploration [1,2,3]. Space-borne electronics can be bombarded by high-energy particles and thus be afflicted with adverse radiation effects, including the single event effect (SEE), total ionizing dose (TID) effect, displacement damage (DD) effect, and space electrostatic discharge (SESD) effect, further triggering off diverse soft and hard errors to the electronic systems [4,5]. Among miscellaneous electronic devices, SiGe electronics have been adapted for various electronic and photonic applications, such as heterojunction bipolar transistors (HBTs), far-infrared photodetectors, and light-emitting diodes [6,7,8]. Leveraging the synergistic effects of band structure and strain engineering, SiGe HBTs can achieve controllable bandgap, increased current gain, and exceptional high-frequency characteristics, as well as inherent compatibility to traditional Si technology, enabling them to be highly suitable for constituting monolithic integrated circuits. Especially, serving as radio-frequency front-end low-noise amplifiers, SiGe HBTs and their bipolar complementary metal oxide semiconductor (BiCMOS) devices can achieve high-speed data transmission with high output power, which de facto renders them promising for high-frequency wireless communications of space missions [9,10,11]. As such, it is indispensable to explore potential radiation damage effects of SiGe-based electronics in the space radiation environment.

Over the past three decades, it has been proved that SiGe HBTs can afford much resistance to Mrad(Si)-level TID effects [12,13,14,15]. Nonetheless, it is noted that SiGe HBTs can be susceptible to SEEs and DD effects [16,17,18,19,20]. Although many efforts have been made on exploring diverse SEEs of SiGe HBTs, few investigations were conducted exclusively on the DD effects. So far, researchers have found that the DD effects of SiGe HBTs can be prevalent under radiation of neutrons, protons, heavy ions, and mixed particles [17,21,22,23]. Sotskov et al. have concluded that SiGe HBTs can suffer from DD effects, typically leading to an increase in the base current and a decrease in the direct current gain, when they are exposed to high-fluence neutrons up to 10^14^ cm^−2^ [10,17]. Sun et al. have revealed that the direct current characteristics of SiGe HBTs can deteriorate owing to the interface traps and DD in the bulk region when the heavy ion radiation of 25 MeV Si ions and 20 MeV Br ions reaches the fluence of 10^10^ cm^−2^ [12,21]. In addition, in a mixed radiation field of neutrons and gamma rays, SiGe HBTs can be damaged synergistically by TID effects and DD effects, and the DD effects sourcing from neutrons may dominate the performance degradation of SiGe HBTs under the low-level injection [22]. These findings demonstrate the susceptibility of SiGe HBTs to DD effects, while the underlying mechanism behind the DD effects is still under investigation.

Since the DD effects stem from defect states, it is essential to probe the defect evolution in fundamental electronic materials under energetic particles. As an efficient atom-scale computation method, molecular dynamics (MD) simulations have been employed to investigate the defect evolution in multiple semiconductors during the collision cascade, such as Si [24,25], Ge [26,27], GaAs [28,29], GaN [30,31], SiC [32,33], and SiGe [34,35,36]. Meanwhile, researchers have also explored the radiation damage of many layered composites and heterostructures via MD simulations [37,38,39,40,41,42]. For instance, Zong et al. have probed the defect evolution of NiFe-graphene nanocomposites and concluded that the interface can act as a defect sink and adsorb interstitials, therefore providing favorable radiation resistance [40]. Huang et al. have investigated the radiation damage and the heat conduction of 3C-SiC/graphene composites, revealing that after the collision cascade induced by 1.5 keV C primary knock-on atom (PKA), the radiation resistance and the thermal conductivity of the composite can be enhanced [42]. Besides, Jiang et al. have employed ab initio MD simulations to examine radiation behaviors of the Si/Ge superlattice, demonstrating that the Si/Ge superlattice is endowed with higher radiation tolerance than bulk Si [41]. These works verify that the DD inside layered materials can differ from that of bulk materials owing to the interface. In this sense, it is imperative to explore the defect evolution of the fundamental SiGe/Si heterostructure in SiGe-based electronics via MD simulations.

The objective of this work was to investigate the primary radiation damage of a strain-engineering-based SiGe/Si heterostructure via MD simulations. The simulation mainly included two parts: (1) independent collision cascades induced by 1 keV, 3 keV, and 5 keV Si PKAs and Ge PKAs; (2) overlapping cascades induced by 3 keV Si PKAs and Ge PKAs. The overlapping cascades were conducted to allow for the defect accumulation during a long-term space operation [43,44]. The defect evolution both in the whole heterostructure and at the heterointerface was evaluated by point defects and cluster defects, while the temperature evolution in the SiGe/Si heterostructure was analyzed by the average lattice temperature and the spatial distribution of lattice temperatures. As such, the atomic-scale mechanism behind the defect evolution and accumulation in a SiGe/Si heterostructure was discussed and revealed. This work will shed light on primary radiation damage of SiGe/Si heterostructures and pertinent radiation hardening techniques of SiGe-based electronics.

## 2. Materials and Methods

The MD simulation was conducted via a classical code of LAMMPS (version: 16 Mar 2018) [45]. A characteristic SiGe/Si heterostructure was constructed by placing a top layer of the Si_0.7_Ge_0.3_ alloy on the Si(001) substrate. The Si_0.7_Ge_0.3_ alloy was assembled by replacing 30% atoms of a Si superlattice randomly with Ge atoms given that Si atoms are completely miscible with Ge atoms in the SiGe alloy [36,46]. The SiGe layer and the Si substrate had a size of 35 × 35 × 11 a_0_^3^ and 35 × 35 × 24 a_0_^3^ (a_0_ is the lattice constant of Si in a diamond structure, 5.43 Å), corresponding to 107,800 and 235,200 atoms, respectively. Herein, the percentage of 30% for the Ge content in the Si_0.7_Ge_0.3_ alloy allowed for the current manufacturing techniques of commercial-off-the-shelf and space-borne SiGe electronic devices (esp. SiGe HBTs), which normally adopt a peak value of less than 30% for the Ge doping in an epitaxial layer of Si substrate [6,9,36,47,48,49]. It is known that the graded Ge content can introduce an electric field in the base region, shortening the base transit time and enhancing the cutoff frequency of SiGe HBTs. As such, a high Ge content is preferred in high-frequency SiGe HBTs, especially for millimeter-wave and tera-hertz transceivers [9,50]. However, given a mismatch of 4.2% between lattice constants of Si and Ge, an overly high Ge content could lead to strain relaxation and misfit dislocation in the epitaxial SiGe layer. As stated in [51], the theoretical critical thickness of a SiGe layer on the Si substrate decreases with the increase in the Ge content, and it is approximately 7.89 nm for the Si_0.7_Ge_0.3_ alloy. In this work, the thickness of the Si_0.7_Ge_0.3_ alloy reached 5.75 nm after the relaxation procedure. As such, the SiGe/Si heterostructure adhered to the strain engineering and maintained the compressive stress in the SiGe alloy. Moreover, atomistic interactions of Si-Si, Ge-Ge, and Si-Ge were described by a hybrid potential function of SW/ZBL, wherein the ZBL was coupled to the SW smoothly by a Fermi-like function [35,36]. With regard to the SW/ZBL potential, the many-body SW potential can represent the cohesive energy, the defect formation, and the melting point of Si and Ge, while the universal ZBL potential undertakes the repulsive force for high-energy collisions in a short range [26,28,52]. The parameters in SW potentials of Si and Ge sourced from [53] and [54], respectively, and the combination between them for the SiGe alloy followed the rule detailed in [55]. The coupling Fermi-like function between SW and ZBL potentials, along with its parameter values for the SiGe alloy, can be readily found in the previous works [27,35,36].

**Figure 1:**
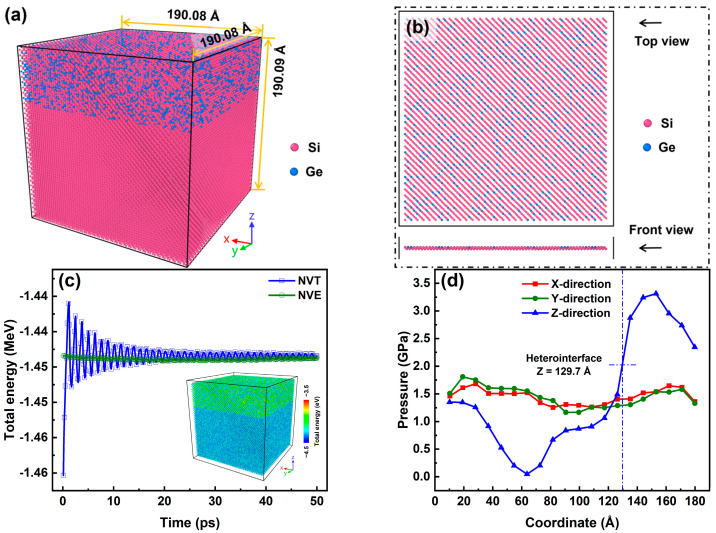
(**a**) A snapshot of the whole heterostructure after the relaxation procedure; (**b**) a snapshot of the heterointerface in the Newtonian region after the relaxation procedure; (**c**) the total energy evolution of the whole heterostructure throughout the relaxation procedure (the inset shows the final total energy distribution in the Newtonian region); (**d**) the final stress distribution of the Newtonian region.

The constructed heterostructure was divided into an inner Newtonian region and an outer thermostat region. The Newtonian region was responsible for the collision cascade, while the thermostat region served the heat conduction between the radiated region and the unirradiated region [56]. The Newtonian region was surrounded by six thermostat slabs with a thickness of 1 a_0_ on the periphery of the heterostructure. Periodic boundary conditions were imposed on the three dimensions of the box-shaped heterostructure. Prior to the simulation of collision cascades, the whole heterostructure was first relaxed under a canonical (NVT) ensemble at 300 K for 50 ps with a timestep of 1 fs to release the internal stress. Then, the Newtonian region was subject to a microcanonical (NVE) ensemble for 50 ps with a timestep of 1 fs to prepare for the collision cascade, while the thermostat region remained in the NVT ensemble of 300 K till the end of the simulation. After the relaxation procedure, the snapshots of the SiGe/Si heterostructure and the heterointerface with two atomic layers are shown in Figure 1a,b. The lattice constant of the relaxed SiGe/Si heterostructure was generally the same as that of Si (5.43 Å). The Ge content was statistically 30.0% in the SiGe alloy, and it approximated to 30.3% on the side of the SiGe alloy at the heterointerface, enabling the Ge content to be 15.2% within the two atomic layers at the heterointerface. Also, as shown in Figure 1c, after the relaxation procedure of 100 ps altogether, the total energy of the whole heterostructure basically stabilized. The final total energy was uniformly distributed both in the SiGe layer and the Si substrate, but the SiGe layer obtained a higher energy level than the Si substrate, which was attributed to the residual stress introduced by the Ge doping. In addition, the stress distribution inside the SiGe/Si heterostructure was determined via the computational method reported in the previous papers [57,58,59]. The pressure tensor of each atom was first calculated and then divided by the atomic volume obtained from the Voronoi analysis in the OVITO software (version: 3.7.10). The stress distribution of the SiGe/Si heterostructure was measured by the average stress in 20 chunks with the same volume along x-direction, y-direction, and z-direction. As shown in Figure 1d, the final stress distribution along the z-direction changed abruptly at the heterointerface owing to the lattice mismatch [58]. By comparison, the final stress distribution was uniform along the x-direction and the y-direction. The compressive stress was almost maximized in the center of the SiGe alloy, while it was basically eliminated in the center of the Si substrate. The phenomenon manifests that the SiGe/Si heterostructure accommodated the compressive stress in the SiGe layer and can emulate a strain-engineering-derived heterostructure.

Two scenarios of collision cascades were carried out, i.e., independent and overlapping collision cascades. As for an independent collision cascade, only one PKA was triggered at the beginning of the collision cascade, while for the overlapping cascades, five PKAs in total were released sequentially; that is, a new PKA would be released each time a previous collision cascade ended. Therefore, except the relaxation procedure before the first collision cascade, intermediate relaxation procedures between five cascades were introduced to the course of overlapping cascades, so that the residual stress of the heterostructure in the previous cascade could be properly eliminated before the beginning of the next cascade. Briefly, in the course of overlapping cascades, as the preceding cascade finished, the resultant heterostructure was equilibrated to control the temperature via the same relaxation procedure (totally 100 ps) of the NVT and NVE as that conducted prior to the first cascade [60]. A typical set of results for five relaxation procedures during the overlapping cascades induced by 3 keV Si PKAs, including the total energy, temperature, and stress in the SiGe/Si heterostructure, are enclosed in Figures S1–S3. During the overlapping cascades, the total energy, temperature, and stress of the whole heterostructure could stabilize before each cascade. It indicates that the residual effects resulted from a previous cascade can be effectively eliminated in a subsequent cascade. Further, as the whole heterostructure was relaxed adequately, a PKA of Si or Ge in the SiGe alloy, located at approximately 20 Å above the heterointerface, was triggered to initiate the collision cascade. A PKA of an independent collision cascade was endowed with an energy level of 1 keV, 3 keV, or 5 keV, while only an energy level of 3 keV was imparted to five PKAs of the overlapping cascades. The PKA was ejected downwards with an angle of 7° against the z-direction to diminish the channeling effect [32,35,42]. Furthermore, based on the area occupied by selected PKAs during the overlapping cascades, the PKA flux was calculated to be 8.55 × 10^23^ PKA∙s^−1^∙cm^−2^ and 5.70 × 10^23^ PKA∙s^−1^∙cm^−2^ for 3 keV Si PKAs and Ge PKAs, respectively. In order to obtain statistically valid results, both independent and overlapping collision cascades were implemented ten times by altering the incidence directions of PKAs, while the positions of corresponding PKAs were fixed. In each run of the overlapping cascades, five PKAs had different incidence directions, and among ten runs of the overlapping cascades, the incidence directions of first PKAs are also different so that the defect evolution in each cascade could have adequate statistical validity. In order to test the statistical validity of the results, the defect numbers in the whole heterostructure for 10 runs and 20 runs of independent collision cascades induced by 5 keV Ge PKAs were compared, since 5 keV Ge PKAs could produce more point defects than the others. The PKA directions and the defect numbers for 10 runs and 20 runs of simulations are presented in Figures S4 and S5. It demonstrates that although the PKA directions for 20 runs could have a larger coverage on the xy-plane than those for 10 runs, the average numbers of six kinds of point defects between 10 runs and 20 runs were close to each other. Therefore, 10 runs of simulations were adopted in this work with the PKA directions as different as possible, so that the results can be statistically reliable. Despite this, the results of the present work could be limited to the partial sampling of the heterostructure and heterointerface because of specific PKA tracks and the random distribution of Ge atoms in the SiGe alloy. Since the collision cascades basically happened in the center of the heterostructure, the results here could neglect the characteristics of other regions of the heterostructure. In order to obtain more effective results, more runs of simulations should be conducted to encompass complete characteristics of the heterostructure and heterointerface.

Also, the effect of the electronic stopping was neglected in the present simulations, since its contribution to the total energy loss of the PKAs was small in the SiGe/Si heterostructure. In fact, as regards the collision cascade induced by a PKA with an energy level less than 10 keV, the electronic stopping has been generally neglected [40,42,61]. Moreover, the accurate electron–phonon coupling parameter for the SiGe alloy has been lacking in the literature. Herein, a Monte Carlo package of Stopping and Range of Ions in Matter (SRIM) was used to probe the role of the electronic stopping in the SiGe/Si heterostructure. The detailed results obtained from the SRIM-2008 software are attached in Table S1. It turned out that the electronic energy loss could contribute at most 17.5% of the total energy loss when 1 keV, 3 keV, and 5 keV Si PKAs and Ge PKAs bombarded pure Si and the Si_0.7_Ge_0.3_ alloy. Meanwhile, the ratios of the electronic energy loss to the nuclear energy loss (S_e_/S_n_) for 1 keV, 3 keV, and 5 keV Si PKAs and Ge PKAs (4.0%~21.2%) were well below those for 20 keV Si PKA (42.8%) and 30 keV Si PKA (55.1%) in SiC [62], which further demonstrates that the electronic stopping could have a small or negligible effect on the number of surviving defects.

The collision cascade was implemented according to a multiple-phase timestep procedure, including the initial phase, intermediate phase, and final phase [32,35,63]. The initial phase lasted for 0.5 ps with timestep of 0.01 fs, the intermediate phase for 50 ps with a timestep of 0.1 fs, and the final phase for 50 ps with a timestep of 1 fs. The defect production and evolution of the whole heterostructure and the heterointerface during the collision cascade was examined via the Wigner–Seitz (WS) defect analysis and the cluster analysis in the OVITO software. The defect analyses at the heterointerface were confined within the two atomic layers across the heterointerface (Figure 1b). As to the WS defect analysis, a lattice site without an atom was regarded as a vacancy, a lattice site with more than one atom was an interstitial, and a lattice site occupied by an atom that had a different atomic type from the original one was an antisite. With respect to the cluster analysis, the second nearest neighbor distance of Si (3.84 Å) was assigned to the cutoff criterion [62,64], and the size of a cluster was measured by the number of atoms contained within. Herein, since the SiGe/Si heterostructure kept the same lattice constant as pure Si (Figure 1a), a unified cutoff criterion of 3.84 Å was adopted for the cluster analysis of the SiGe/Si heterostructure. Besides, the number of vacancies was also employed to denote the number of Frenkel pairs since vacancies and interstitials were produced parallel to each other. Also, the number of Frenkel pairs surviving at the end of the collision cascade was justified by the Norgett–Robinson–Torrens (NRT) model. The calculation method was sourced from previous papers [65,66] and is provided in the Supplementary Information. The recombination ratio of Frenkel pairs was measured by dividing the difference between the maximum and final numbers by the maximum number.

The melting region formed inside the Newtonian region during the collision cascade was identified by the melting points of pure Si and the Si_0.7_Ge_0.3_ alloy, which were obtained via MD simulations based on the solid–liquid coexistence approach [35,67,68]. The melting point was calculated to be the midpoint of the temperature range where the volume decreased sharply. As shown in Figure S6, the melting points of pure Si and the Si_0.7_Ge_0.3_ alloy were calculated to be 1675 K and 1950 K, respectively, which were comparable to previous results (Si: 1653 K; Ge: 2885 K) in MD simulations supported by the same type of potential function [26,35]. As such, when the lattice temperature of any chunk in the SiGe alloy and the Si substrate exceeded the corresponding melting point, the chunk was recognized as a portion of the melting region. In addition, the atomic temperature was derived from the kinetic energy according to an equation *E* = (3/2) × *k*_B_ × *T*, where *E* is the kinetic energy, *k*_B_ is the Boltzmann’s constant, and *T* is the temperature.

In all figures, the error bar denotes the standard deviation of the corresponding data. For visual clarity, sometimes half of the error bar is shown, but this does not alter its meaning.

## 3. Results and Discussion

### 3.1. Defect Evolution of an Independent Collision Cascade

**Figure 2:**
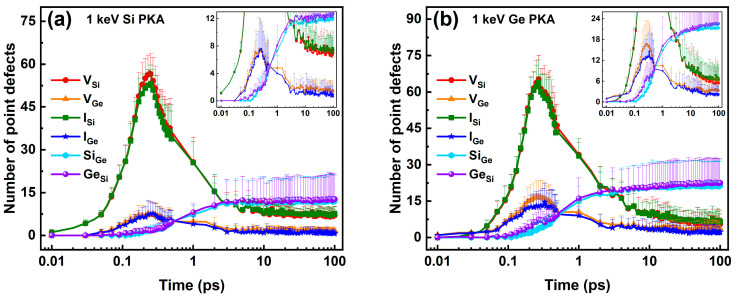

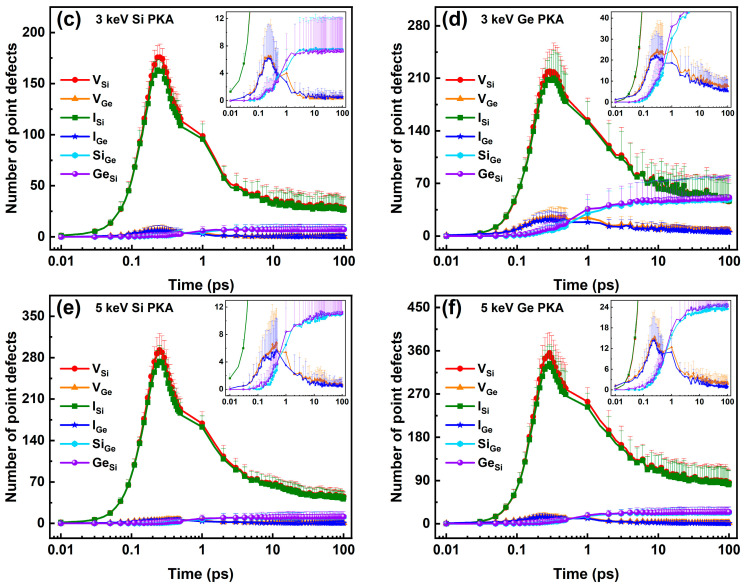
The six types of point defects in the whole heterostructure during independent collision cascades induced by (**a**) 1 keV Si PKA, (**b**) 1 keV Ge PKA, (**c**) 3 keV Si PKA, (**d**) 3 keV Ge PKA, (**e**) 5 keV Si PKA, and (**f**) 5 keV Ge PKA (each inset shows an enlarged detail of the corresponding subfigure).

The numbers of six types of point defects in the whole heterostructure during independent collision cascades induced by 1 keV, 3 keV, and 5 keV Si PKAs and Ge PKAs are shown in Figure 2. Overall, the numbers of the same type of point defects evolved in a similar trend over the course of the collision cascade, regardless of the energy (1 keV, 3 keV, or 5 keV) and type (Si or Ge) of PKAs. Specifically, the numbers of vacancies and interstitials first reached their apexes at about 0.25 ps, then descended, and finally tended to stabilize at 30 ps or so, which echoed with three stages of the collision cascade, i.e., the ballistic stage, the thermal spike stage, and the quenching stage [65,69]. However, the apexes of vacancies and interstitials for Ge PKAs occurred approximately 0.3 ps later than those for Si PKAs; with the PKA energy increasing from 1 keV to 5 keV, the thermal spike stage might last longer, while the quenching stage was postponed. By comparison, the numbers of antisites continued increasing in the first two stages and were inclined to stabilize in the third stage. As the final number of antisites increased at the end of the collision cascades, the time for antisites to begin stabilizing could delay slightly. The discrepancy in the evolution trends between vacancies/interstitials and antisites indicates that the recombination of Frenkel pairs in the thermal spike stage could facilitate the formation of antisites, which was similar to the observations in SiGe alloys and several intermetallic alloys [34,35,70]. Furthermore, in each collision cascade, the number of Si vacancies (V_Si_) was more than that of Ge vacancies (V_Ge_), which was linked to a high percentage of Si atoms in the SiGe/Si heterostructure, including the top SiGe layer and the bottom Si substrate. The ratio of the number of V_Si_ to that of V_Ge_ also became greater for PKAs with more energy, which could reach deeper in the Si substrate of the SiGe/Si heterostructure and displace more Si atoms. A set of snapshots of atomic displacements during the initial phase of an independent collision cascade induced by a 3 Ge PKA are presented in Figure S7. At first, the Ge PKA had a kinetic energy of 2994.77 eV. After 0.02 ps, the Ge PKA was left an energy level of 2125.60 eV owing to the atomic collisions. At 0.03 ps, a Si atom at the heterointerface was struck out by the Ge PKA and obtained an energy level of 267.74 eV. Then, the Ge PKA passed through the heterointerface and continued to collide with other atoms in the Si substrate. Since 0.05 ps, a number of atoms at the heterointerface were collided by secondary knock-on atoms coming from the regions below or above the heterointerface, and at 0.2 ps, a mass of atomic collisions already happened in the center of the SiGe/Si heterostructure.

**Figure 3:**
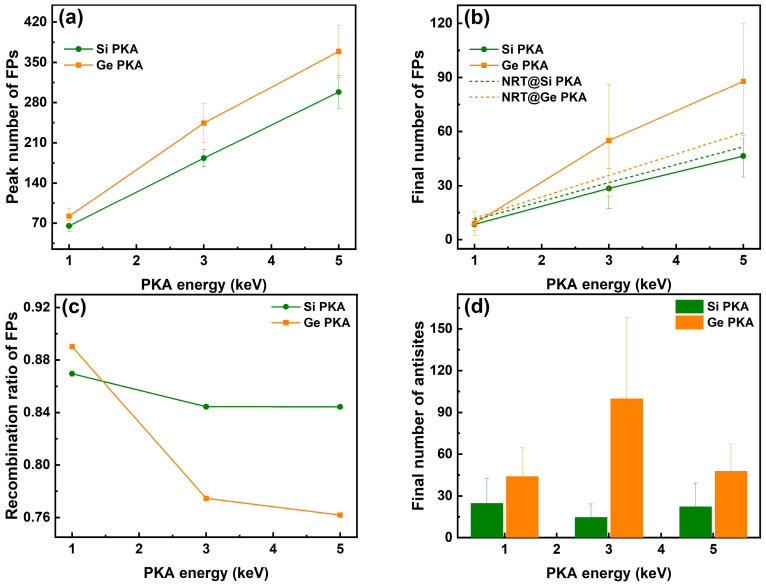
The peak numbers of Frenkel pairs (FPs) (**a**), the final numbers of FPs as well as the results of the NRT model (**b**), the recombination ratio of FPs (**c**), and the final number of antisites (**d**) in the whole heterostructure during independent collision cascades induced by 1 keV, 3 keV, and 5 keV Si PKAs and Ge PKAs.

As shown in Figure 3, for the same type of PKA, the peak and final numbers of Frenkel pairs (V_Si_ + V_Ge_) were proportional to the PKA energy, while for the same PKA energy, Ge PKAs secured higher peak and final numbers of Frenkel pairs than Si PKAs, and this difference between Si PKAs and Ge PKAs could be more prominent as the PKA energy increased from 1 keV to 5 keV. It suggests that PKAs with higher energy and greater atomic mass could result in more Frenkel pairs, which aligned with the NRT model and previous research on collision cascades of SiC and SiGe alloys [32,35,71]. Although the final numbers of Frenkel pairs for 1 keV, 3 keV, and 5 keV Si PKAs as well as 1 keV Ge PKAs were comparable to the results of the NRT model, the final numbers of Frenkel pairs for 3 keV and 5 keV Ge PKAs were much higher than those of the NRT model. Meanwhile, the recombination ratio of Frenkel pairs for the same type of PKA decreased with the PKA energy increasing from 1 keV to 5 keV, and the decreasing trend was more noticeable for Ge PKAs than Si PKAs. The recombination ratios of Frenkel pairs for 3 keV and 5 keV Ge PKAs were also lower than those for 3 keV and 5 keV Si PKAs, respectively, which led to more Frenkel pairs for 3 keV and 5 keV Ge PKAs at the end of collision cascades. Furthermore, the low recombination ratios for 3 keV and 5 keV Ge PKAs were related to the melting region formed during the collision cascades, hindering the recombination of Frenkel pairs in the SiGe/Si heterostructure. In contrast, the final numbers of antisites (Si_Ge_ + Ge_Si_) were not proportional to the PKA energy. The final numbers of antisites for 1 keV Si PKAs and Ge PKAs approached those of 5 keV Si PKAs and Ge PKAs, respectively. However, the final numbers of antisites for 3 keV Ge PKAs were apparently higher than those for 1 keV and 5 keV Ge PKAs, while those for 3 keV Si PKAs were slightly lower than those for 1 keV and 5 keV Si PKAs. Since the antisites were mainly derived from the top SiGe layer of the SiGe/Si heterostructure, a shorter PKA range could lead to more energy dissipated in the SiGe alloy and consequently generate more antisites after the collision cascade. Therefore, the final number of antisites could rely little on the initial PKA energy but much on the PKA track, and accordingly it was the tracks of 3 keV Ge PKAs that probably lied nearby the heterointerface, dissipated more energy, and produced more antisites. Besides, a high standard deviation of final numbers of antisites for 3 keV Ge PKAs connotes that the number of antisites strongly depended on the incidence directions of PKAs.

**Figure 4:**
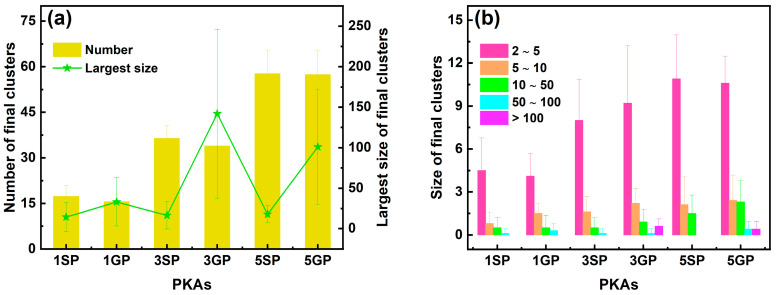
The number and the largest size of final clusters (**a**) along with the size distribution of final clusters (**b**) in the whole heterostructure during independent collision cascades induced by 1 keV, 3 keV, and 5 keV Si PKAs and Ge PKAs (1SP, 1GP, 3SP, 3GP, 5SP, and 5GP represent 1 keV Si PKA, 1 keV Ge PKA, 3 keV Si PKA, 3 keV Ge PKA, 5 keV Si PKA, and 5 keV Ge PKA, respectively; this annotation is available for the same notations in other figures).

In addition to the point defects, the number and size of final clusters in the whole heterostructure during independent collision cascades are shown in Figure 4. Irrespective of PKA types, the number of final clusters increased with the rising PKA energy, and at the same PKA energy, the numbers of final clusters were comparable for two types of PKAs. The largest sizes of final clusters were close for 1 keV, 3 keV, and 5 keV Si PKAs, whereas those were the greatest for 3 keV Ge PKAs, followed by 5 keV Ge PKAs and 1 keV Ge PKAs. Also, at the same PKA energy, the largest sizes of final clusters for Ge PKAs were greater than those for Si PKAs, especially for 3 keV and 5 keV PKAs. Besides, the largest size of final clusters for 3 keV Ge PKAs somehow matched with their final number of antisites; that is, both of them were the greatest among all PKAs, which implies that the antisites significantly contributed to the final clusters of 3 keV Ge PKAs. Meanwhile, with the PKA energy increasing from 1 keV to 5 keV, all sizes of final clusters were inclined to accumulate, especially small-sized final clusters, and the final clusters with a larger size might emerge for Ge PKAs. Most of final clusters had a size ranging from 2 to 5, and only 3 keV and 5 keV Ge PKAs could averagely generate less than one final cluster with a size over 100, which was consistent with the largest size of final clusters for 3 keV and 5 keV Ge PKAs. These results indicate that in comparison with Si PKAs, Ge PKAs were prone to generate more and larger clusters in the whole heterostructure at the end of the collision cascade. It was consistent with previous findings in SiGe alloys [34,35]; that is, as compared to Si PKAs with the same energy, Ge PKAs could produce dense and compact collision cascades and retain large cluster defects.

**Figure 5:**
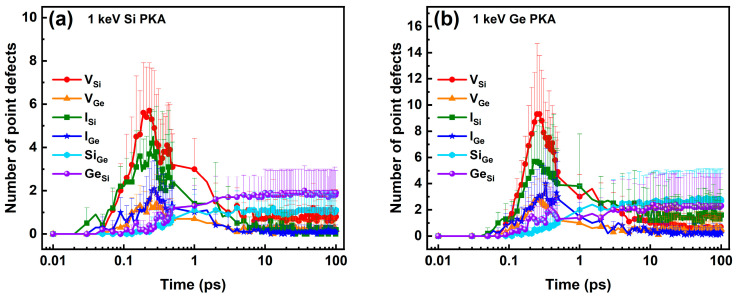

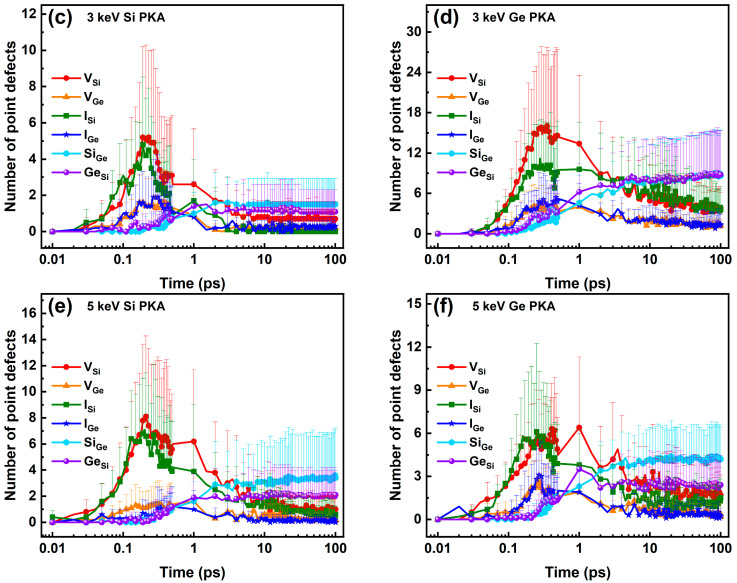
The six types of point defects at the heterointerface during independent collision cascades induced by (**a**) 1 keV Si PKA, (**b**) 1 keV Ge PKA, (**c**) 3 keV Si PKA, (**d**) 3 keV Ge PKA, (**e**) 5 keV Si PKA, and (**f**) 5 keV Ge PKA.

Likewise, the defect evolution at the heterointerface of the SiGe/Si heterostructure was analyzed. Figure 5 presents the numbers of six types of point defects at the heterointerface during independent collision cascades induced by 1 keV, 3 keV, and 5 keV Si PKAs and Ge PKAs, which basically evolved in a similar trend to those in the whole heterostructure, respectively. Notably, throughout the collision cascade, the numbers of six types of point defects at the heterointerface were significantly fewer and fluctuated more obviously than those in the whole heterostructure.

**Figure 6:**
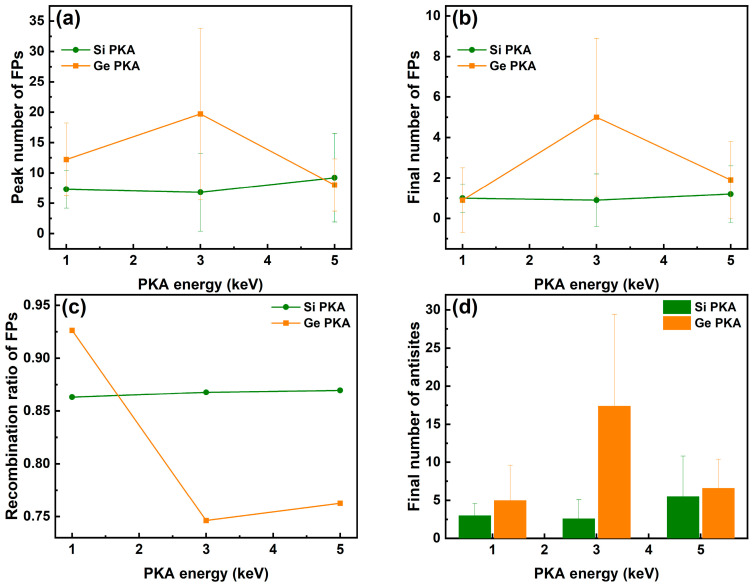
The peak numbers of FPs (**a**), the final numbers of FPs (**b**), the recombination ratio of FPs (**c**), and the final numbers of antisites (**d**) at the heterointerface during independent collision cascades induced by 1 keV, 3 keV, and 5 keV Si PKAs and Ge PKAs.

As shown in Figure 6, with the PKA energy rising from 1 keV to 5 keV, the peak numbers of Frenkel pairs between Si PKAs kept close, while 3 keV Ge PKAs produced more peak Frenkel pairs than 1 keV Ge PKAs and 5 keV Ge PKAs. A similar phenomenon happened to the final numbers of Frenkel pairs. Meanwhile, the recombination ratio of Frenkel pairs for 1 keV Ge PKAs was higher than that for 1 keV Si PKAs, which made their final numbers of Frenkel pairs close to each other. The recombination ratios of Frenkel pairs for 3 keV and 5 keV Ge PKAs were apparently lower than those for 3 keV and 5 keV Si PKAs, respectively, which was similar to the phenomenon observed in the whole heterostructure. In addition, the final numbers of antisites at the heterointerface were similar to those in the whole structure, and the final numbers of antisites for 3 keV Ge PKAs were still the greatest among all cases. Herein, different from that in the whole heterostructure, the number of Frenkel pairs at the heterointerface was non-proportional to the PKA energy. The PKAs could penetrate deeper in the Si substrate with the PKA energy rising from 1 keV to 5 keV, which caused less energy dissipated in the SiGe layer and few defects produced at the heterointerface. Therefore, the tracks of 3 keV Ge PKAs could settle around the heterointerface and bring about considerable point defects at the heterointerface. Still, for each collision cascade, the number of V_Si_ was more than that of V_Ge_ since the two layers of the heterointerface contained more Si atoms than Ge atoms.

**Figure 7:**
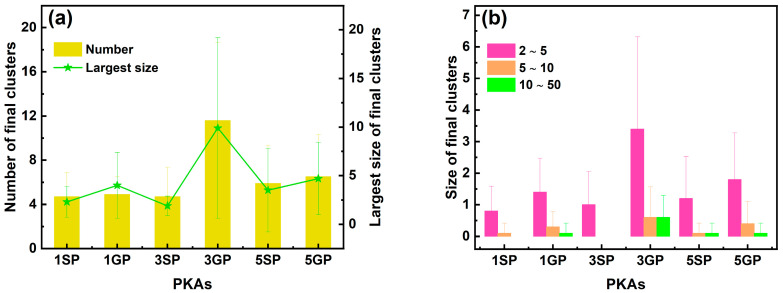
The number and the largest size (**a**) along with the size distribution (**b**) of final clusters at the heterointerface during independent collision cascades induced by 1 keV, 3 keV, and 5 keV Si PKAs and Ge PKAs.

As shown in Figure 7, the final clusters for 1 keV, 3 keV, and 5 keV Si PKAs and Ge PKAs at the heterointerface were fewer and smaller than those in the whole heterostructure, respectively. Unlike those in the whole heterostructure, the numbers of final clusters at the heterointerface were not proportional to the PKA energy, and those for 3 keV Ge PKAs were the greatest among all cases. The largest sizes of final clusters between PKAs shared a similar pattern to that in the whole heterostructure, and 3 keV Ge PKAs produced the largest final clusters. The clusters with a size of 2~5 still dominated the number of final clusters, and all sizes of final clusters for 3 keV Ge PKAs were more than those for the other PKAs, respectively. Overall, Ge PKAs were also prone to generate more and larger clusters at the heterointerface than Si PKAs, which further demonstrates that 3 keV Ge PKAs could dissipate more energy and produce more defects at the heterointerface.

**Figure 8:**
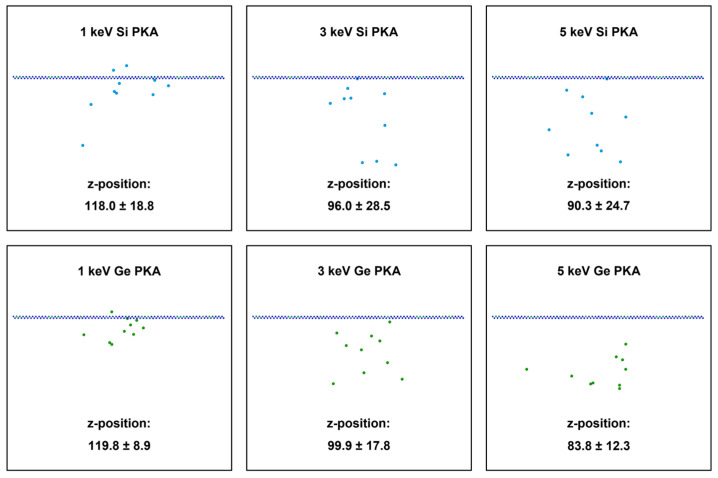
The final z-positions of PKAs in the SiGe/Si heterostructure during independent collision cascades induced by 1 keV, 3 keV, and 5 keV Si PKAs and Ge PKAs (two rows of orderly small balls represent atoms at the heterointerface, while other scattered big balls denote PKAs; this annotation is applicable to similar figures).

The defect production was directly linked to energy dissipation and transfer along the PKA track. The energy dissipated by PKAs could transfer to surrounding lattice atoms and lead to the vibration and displacement of the atoms. In terms of the whole heterostructure, all the PKA energy could be exhausted in the Newtonian region, which enabled the defect number to be proportional to the PKA energy. In contrast, at the heterointerface, the energy dissipation of PKAs was confined in two atomic layers and could be significantly affected by specific PKA tracks. In order to evaluate the energy dissipation of PKAs at the heterointerface, the final z-positions of 1 keV, 3 keV, and 5 keV Si PKAs and Ge PKAs in the SiGe/Si heterostructure were measured, as displayed in Figure 8. Generally, most PKAs could penetrate through the heterointerface and reach deep in the Si substrate, while few bounced back into the SiGe alloy. As the PKA energy increased from 1 keV to 5 keV, a PKA could reach deeper in the Si substrate and settle at a lower z-position. As regards the average value of final z-positions, 1 keV and 3 keV Ge PKAs could settle at slightly higher z-positions than 1 keV and 3 keV Si PKAs, respectively, while 5 keV Ge PKAs reached deeper than 5 keV Si PKAs. However, it was noted that the final z-positions of Si PKAs might span a larger region than those of Ge PKAs, as suggested by greater standard variations in final z-positions for Si PKAs. In other words, final Si PKAs were more dispersed along the z-direction in the SiGe/Si heterostructure than final Ge PKAs. In addition, the nearest distance from the heterointerface (z-position: 129.7) along the z-direction was acquired by 1 keV Ge PKAs, followed by 1 keV Si PKAs, 3 keV Ge PKAs, 3 keV Si PKAs, 5 keV Si PKAs, and 5 keV Ge PKAs. As the final z-positions of PKAs lied closer to the heterointerface, more PKA energy could be dissipated at the heterointerface, producing more point defects and clusters. Despite residing the nearest distance from the heterointerface, 1 keV Ge PKAs was limited to insufficient energy and incapable of generating many defects. In contrast, with the second nearest distance from the heterointerface, 3 keV Ge PKAs were endowed with more energy and could produce considerable defects at the heterointerface, including Frenkel pairs, antisites, and clusters. Besides, for all PKAs, the antisites were more prevalent than vacancies and interstitials at the heterointerface, which was also quite noticeable for 3 keV Ge PKAs. Therefore, in comparison with other PKAs, 3 keV Ge PKAs could be detrimental to the functional role of the SiGe/Si heterostructure in SiGe-based electronics. These results demonstrate that the defect production at the heterointerface was strongly dependent on specific PAK tracks, but little on the initial PKA energy.

### 3.2. Defect Evolution of the Overlapping Cascades

**Figure 9:**
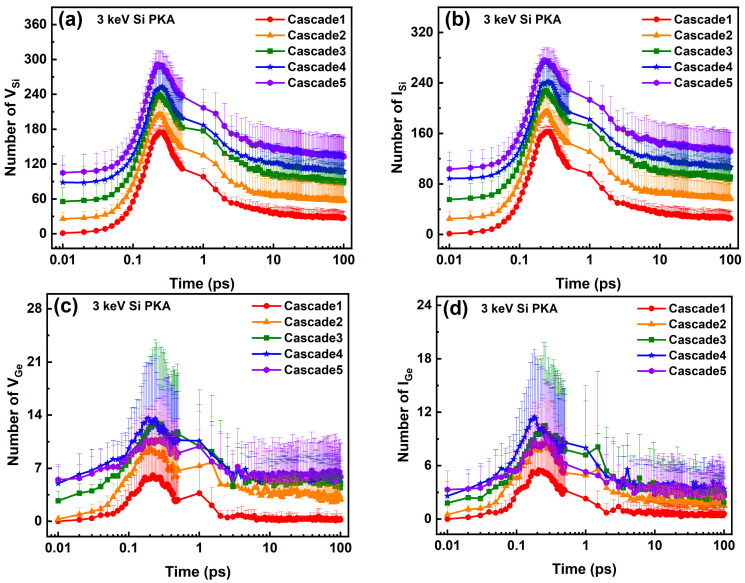

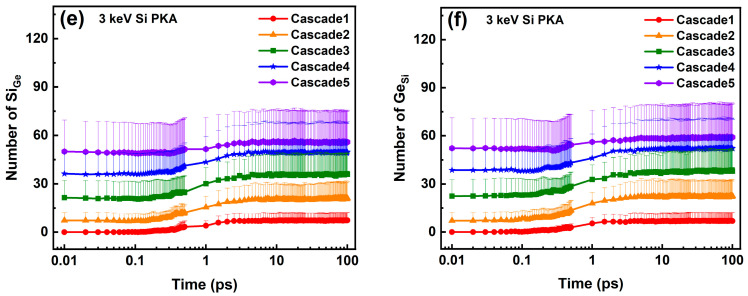
The six types of point defects of (**a**) V_Si_, (**b**) I_Si_, (**c**) V_Ge_, (**d**) I_Ge_, (**e**) Si_Ge_, and (**f**) Ge_Si_ in the whole heterostructure during the overlapping cascades induced by 3 keV Si PKAs.

During the overlapping cascades, five PKAs successively brought about five collision cascades. The point defect evolution of the whole heterostructure for five cascades induced by 3 keV Si PKAs is presented in Figure 9. The evolution trends of six types of point defects in the whole heterostructure for all five cascades were akin to those of independent collision cascades induced by 3 keV Si PKAs, respectively. The numbers of six types of point defects for the first cascade were reasonably equivalent to those for independent collision cascades induced by 3 keV Si PKAs, respectively. Moreover, the numbers of V_Si_, Si interstitials (I_Si_), Si antisites (Si_Ge_), and Ge antisites (Ge_Si_) could increase progressively from the first to the fifth cascades. However, the numbers of V_Ge_ and Ge interstitials (I_Ge_) merely continued to rise in the first four cascades but fell for the fifth cascade. In the thermal spike phase, the numbers of V_Ge_ and I_Ge_ for the fifth cascade lied between those for the second and the third cascades, respectively, while at the end of the collision cascades, they were close to those for the fourth cascade, respectively. Meanwhile, the point defect evolution of the whole heterostructure for five cascades induced by 3 keV Ge PKAs is enclosed in Figure S8. The numbers of V_Si_, I_Si_, Si_Ge_, and Ge_Si_ for 3 keV Ge PKAs also increased from the first to the fifth cascades, which was the same as the observations for 3 keV Si PKAs. However, the numbers of V_Ge_ and I_Ge_ could increase gradually from the first to fifth cascades, except the fourth cascade. Specifically, at the end of the collision cascades, the numbers of V_Ge_ and I_Ge_ for the fourth cascade were comparable to those for the third cascade, respectively, while those for the fifth cascade could surpass those for the other cascades, respectively.

Overall, during the overlapping cascades of 3 keV Si PKAs and Ge PKAs, the numbers of V_Si_, I_Si_, Si_Ge_, and Ge_Si_ in the whole heterostructure could accrue from the first to the fifth cascades, while the numbers of V_Ge_ and I_Ge_ in the whole heterostructure continued to increase only in the first three cascades and might cease to grow in the course of the fourth or the fifth cascades. The anomaly in the numbers of V_Ge_ and I_Ge_ could be attributed to the annihilation of point defects. As stated in [72], with regard to 12 sequential cascades in Si, the coordination defects increase proportionally in the first four cascades, but since the fifth cascade, the number of coordination defects in each cascade has decreased by 36% as compared to that in an independent collision cascade. It is concluded that the amorphous structure formed in the previous cascades consumes the kinetic energy of subsequent PKAs, thereby reducing the defect production in the ensuing cascades. Also, as reported by Zong et al. [40], the numbers of vacancies and interstitials produced in four NiFe-graphene nanocomposites were not proportional to the cascade number during the overlapping cascades containing ten cascades. With regard to four kinds of materials, the numbers of vacancies and interstitials could increase in the first four cascades but begin to fall and rise in the subsequent cascades. Rymzhanov et al. also found that when the irradiated region was overlapped by a subsequent swift heavy ion (SHI) trajectory, the annealing of existing defects could happen owing to the elevated lattice temperature in the vicinity of the SHI trajectory [73]. Therefore, it was inferred that after the first three cascades, the subsequent cascades could lead to the annihilation of V_Ge_ and I_Ge_ owing to the overlapping damage regions.

**Figure 10:**
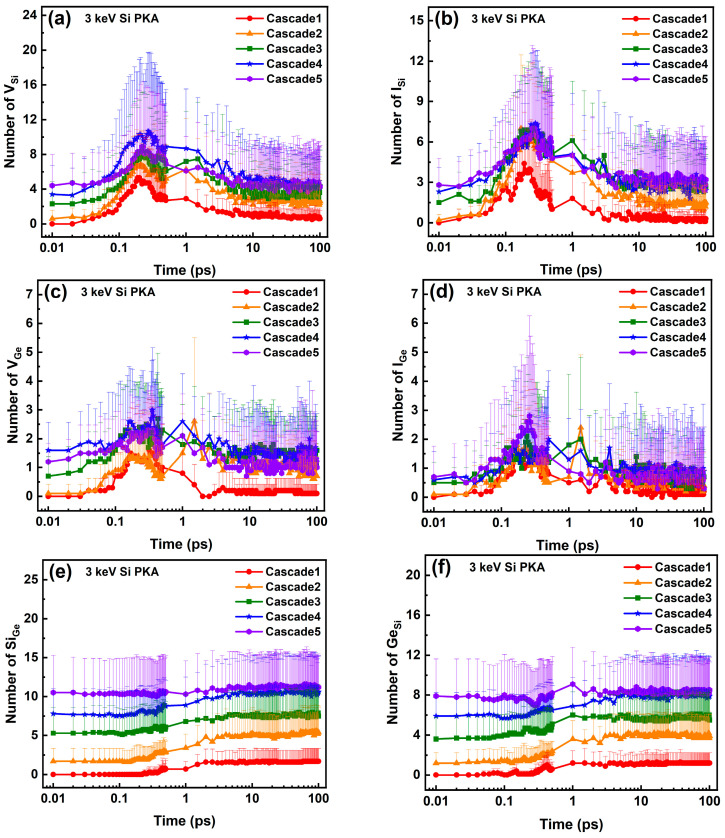
The six types of point defects of (**a**) V_Si_, (**b**) I_Si_, (**c**) V_Ge_, (**d**) I_Ge_, (**e**) Si_Ge_, and (**f**) Ge_Si_ at the heterointerface during the overlapping cascades induced by 3 keV Si PKAs.

Accordingly, the point defect evolution of the heterointerface for five cascades induced by 3 keV Si PKAs is presented in Figure 10. Generally, the evolution trends of six types of point defects for all five cascades at the heterointerface were analogous to those of independent collision cascades induced by 3 keV Si PKAs, respectively, and they were accompanied by significant fluctuation during each cascade of the overlapping cascades, especially V_Ge_ and I_Ge_. Among six types of point defects, only the numbers of Si_Ge_ and Ge_Si_ increased from the first to the fifth cascades, although the increment was small from the fourth to the fifth cascades. The numbers of V_Si_ and I_Si_ for the fifth cascade dropped to a lower level than those for the fourth cascade during the thermal spike phase, respectively, while they were still comparable to those for the fourth cascade at the end of the collision cascades. Besides, the numbers of V_Ge_ and I_Ge_ for the third, the fourth, and the fifth cascades could intertwine notably during the second and third phases of each cascade, respectively. After the collision cascades, the numbers of V_Ge_ and I_Ge_ for the fifth cascade were lower than those for the fourth cascade, respectively. The point defect evolution of the heterointerface for five cascades induced by 3 keV Ge PKAs is also provided in Figure S9. The evolution trends of six types of point defects for five cascades at the heterointerface for 3 keV Ge PKAs were similar to those for 3 keV Si PKAs, respectively. Particularly, the numbers of antisites could increase distinctly from the first to the fifth cascades. The numbers of V_Si_, V_Ge_, I_Si_, and I_Ge_ for the fourth cascade could drop to a comparable level to those for the third cascade in the course of the collision cascades, while those for the fifth cascade could still exceed those for the previous cascades after the collision cascades, which was similar to the phenomenon in the whole heterostructure for 3 keV Ge PKAs. That is, for 3 keV Ge PKAs, all six types of point defects in the fifth cascade were the most among five cascades of the overlapping cascades.

Generally, the defect production and evolution at the heterointerface shared similar characteristics with that in the whole heterostructure. The numbers of six types of point defects of the whole heterostructure and the heterointerface could accumulate in a degree after five cascades of the overlapping cascades, and all of them were proportional to the cascade number in the first three cascades. By comparison, 3 keV Ge PKAs could accumulate more point defects than 3 keV Si PKAs both in the whole heterostructure and at the heterointerface.

**Figure 11:**
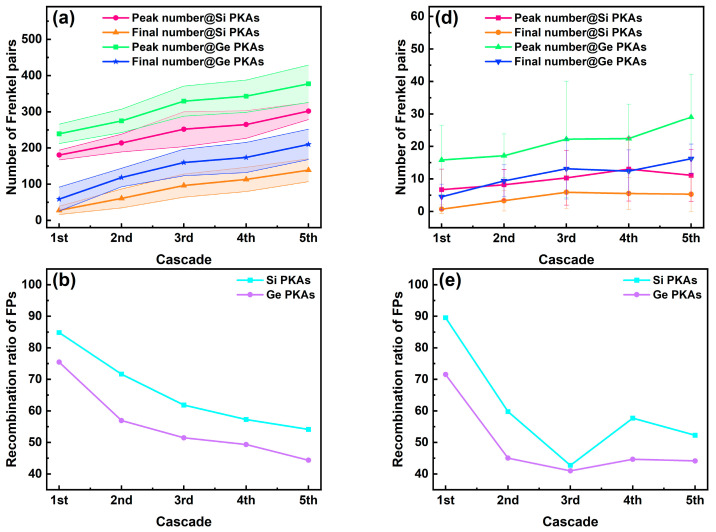

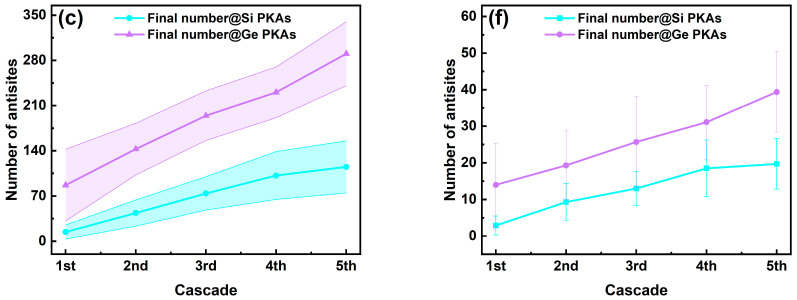
The peak and final numbers of FPs, the recombination ratios of FPs, and the final number of antisites in the whole heterostructure (**a**–**c**) and at the heterointerface (**d**–**f**).

The peak and final numbers of Frenkel pairs, the recombination ratios of Frenkel pairs, and the final numbers of antisites in the whole heterostructure and at the heterointerface during the overlapping cascades induced by 3 keV Si PKAs and Ge PKAs are shown in Figure 11. In terms of the whole heterostructure, the peak and final numbers of Frenkel pairs as well as the final numbers of antisites for 3 keV Si PKAs and Ge PKAs could increase continually from the first to the fifth cascades. At the end of each cascade, 3 keV Ge PKAs could produce more Frenkel pairs and antisites than 3 keV Si PKAs in the whole heterostructure, which was akin to the results of independent collision cascades. Also, the recombination ratios of Frenkel pairs in the whole heterostructure decreased as the cascade number increased. In contrast, at the heterointerface, the peak and final numbers of Frenkel pairs for 3 keV Si PKAs and Ge PKAs merely continued to grow in the first three cascades. As for 3 keV Si PKAs, the peak number of Frenkel pairs in the fifth cascade was lower than that in the fourth cascade, and the final numbers of Frenkel pairs in the fourth and fifth cascades were also slightly lower than that in the third cascade. With respect to 3 keV Ge PKAs, the peak number of Frenkel pairs in the fourth cascade was quite close to that in the third cascade, while the final number of Frenkel pairs in the fourth cascade was slightly lower than that in the third cascade. Besides, the recombination ratios of Frenkel pairs at the heterointerface continued to decrease for the first third cascades and tended to fluctuate in the fourth and fifth cascades. Despite that, from the first to the fifth cascades, the final numbers of antisites still kept increasing at the heterointerface, which was similar to the phenomenon in the whole heterostructure. Basically, these results reveal that during the overlapping cascades induced by 3 keV Si PKAs and Ge PKAs, the Frenkel pairs at the heterointerface could annihilate in the fourth or the fifth cascades.

**Figure 12:**
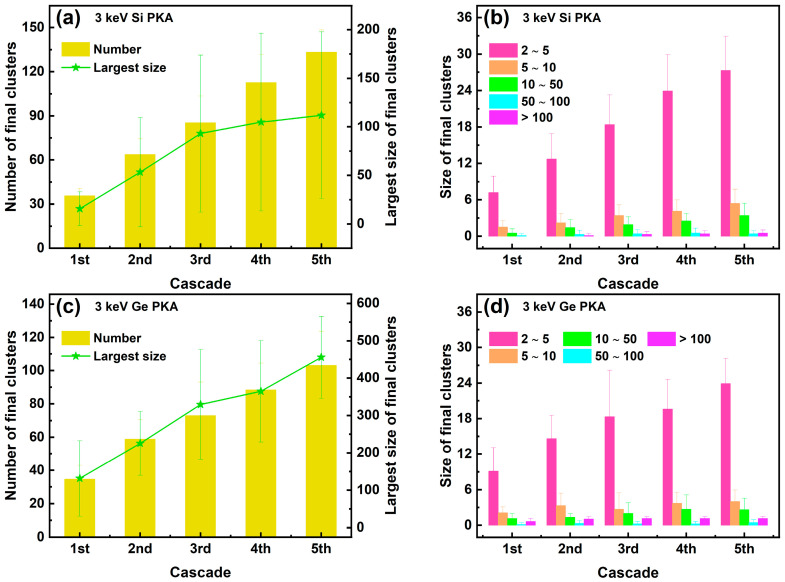
The number and the largest size along with the size distribution of final clusters in the whole heterostructure during the overlapping cascades induced by 3 keV Si PKAs (**a**,**b**) and Ge PKAs (**c**,**d**).

The number and size of final clusters in the whole heterostructure during the overlapping cascades induced by 3 keV Si PKAs and Ge PKAs are shown in Figure 12. The number and size of final clusters in the first cascade of 3 keV Si PKAs and Ge PKAs were equivalent to those in independent collision cascades of 3 keV Si PKAs and Ge PKAs, respectively. Both the number and the largest size of final clusters kept increasing from the first to the fifth cascades, especially those for 3 keV Ge PKAs. Also, all sizes of final clusters for 3 keV Si PKAs and Ge PKAs attempted to grow throughout the overlapping cascades, particularly small clusters with a size of 2~5. By comparison, 3 keV Ge PKAs might generate fewer but larger final clusters than 3 keV Si PKAs, which matched with the results of independent collision cascades induced by 3 keV Si PKAs and Ge PKAs.

**Figure 13:**
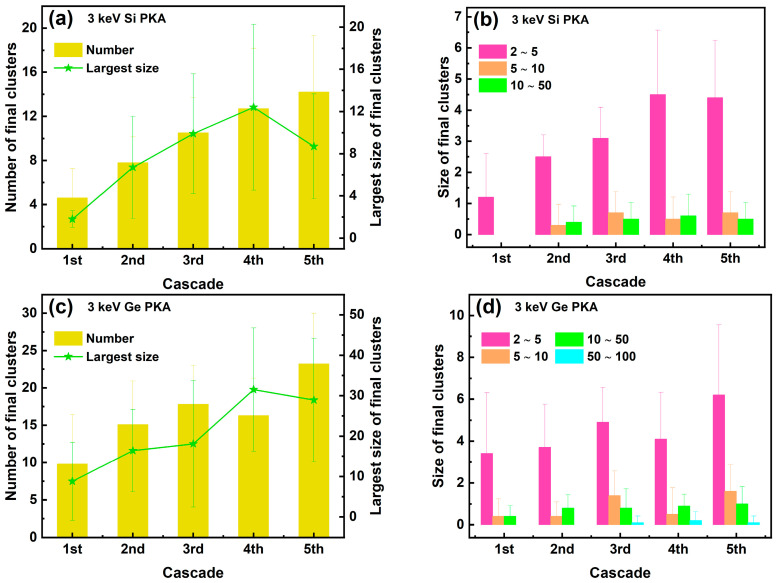
The number and the largest size, along with the size distribution of final clusters at the heterointerface during the overlapping cascades induced by 3 keV Si PKAs (**a**,**b**) and Ge PKAs (**c**,**d**).

Meanwhile, the number and size of final clusters at the heterointerface during the overlapping cascades induced by 3 keV Si PKAs and Ge PKAs are shown in Figure 13. The number of final clusters for 3 keV Si PKAs could still increase throughout the overlapping cascades, while that for 3 keV Ge PKAs only grew steadily in the first three cascades. The largest sizes of final clusters for 3 keV Si PKAs and Ge PKAs increased continuously in the first four cascades but decreased for the fifth cascade. Moreover, all sizes of final clusters could cease to grow for the fourth or the fifth cascades. Despite that, 3 keV Ge PKAs could still produce more and larger final clusters at the heterointerface than 3 keV Si PKAs. It resonated with the results of point defects at the heterointerface. 3 keV Ge PKAs produced more final point defects (especially antisites) than 3 keV Si PKAs, which thus contributed to the formation of final clusters.

**Figure 14:**
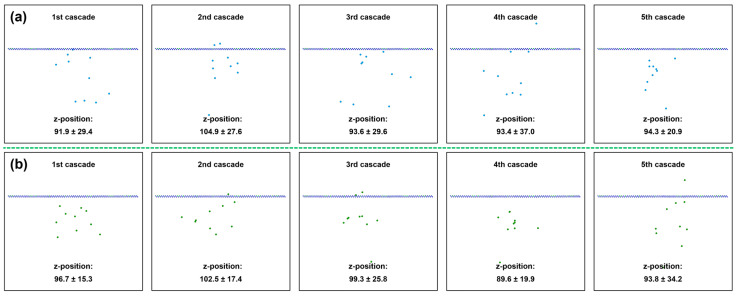
The final z-positions of PKAs in the SiGe/Si heterostructure during the overlapping cascades: (**a**) 3 keV Si PKAs; (**b**) 3 keV Ge PKAs.

Furthermore, the snapshots of final z-positions of 3 keV Si PKAs and Ge PKAs in the SiGe/Si heterostructure for each cascade of the overlapping cascades are presented in Figure 14. As regards five cascades, the final z-positions of 3 keV Si PKAs and Ge PKAs were statistically close, although those in the second cascades were, on average, higher than those in the other cascades. It suggests that the final z-positions of 3 keV Si PKAs and Ge PKAs during the overlapping cascades were basically equivalent to those in independent collision cascades and trivially affected by the damaged structure caused by the collision cascades. The apparent difference in the final z-positions of 3 keV Si PKAs and Ge PKAs between cascades could possibly be accounted for by random incidence directions of PKAs. These results demonstrate that the defect evolution in each cascade was relatively standalone so that the defect number might be proportional to the cascade number, as reflected by the results of point defects and final clusters in the whole heterostructure. Notably, around the heterointerface, the spatial distribution of final z-positions of 3 keV Si PKAs and Ge PKAs varied between cascades. In the first four cascades, the number of 3 keV Si PKAs near the heterointerface were higher than that in the fifth cascade. However, in the fourth cascade, the number of 3 keV Ge PKAs around the heterointerface were few as compared to that in the other cascades. As the distance between the final z-positions of PKAs and the heterointerface increased, less PKA energy could be dissipated at the heterointerface, resulting in fewer point defects. The evolution of the final z-positions of 3 keV Si PKAs and Ge PKAs around the heterointerface could be linked to the discrepancy of the numbers of point defects and final clusters at the heterointerface between cascades, especially the numbers of Frenkel pairs. These results further verify that the PKA energy dissipated around the heterointerface could affect the evolution of point defects at the heterointerface.

### 3.3. Temperature Evolution of Independent and Overlapping Collision Cascades

**Figure 15:**
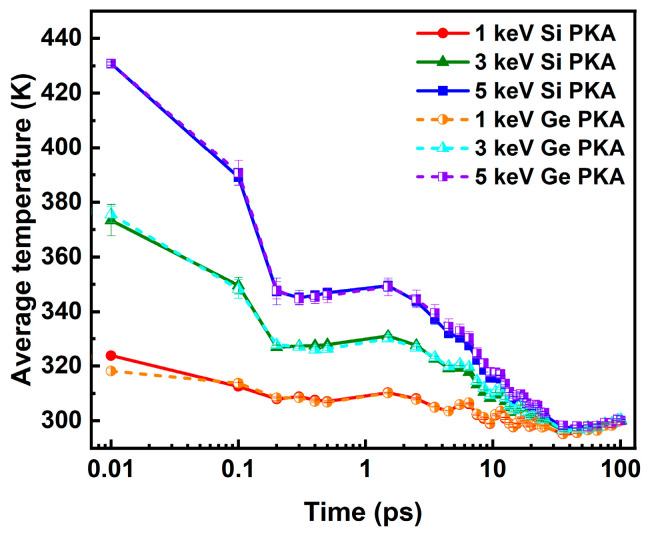
The average lattice temperatures of the Newtonian region in the SiGe/Si heterostructure during independent collision cascades induced by 1 keV, 3 keV, and 5 keV Si PKAs and Ge PKAs.

Along with the defect evolution, the lattice temperature of the SiGe/Si heterostructure could evolve during the collision cascade. The average lattice temperatures of the Newtonian region during independent collision cascades induced by 1 keV, 3 keV, and 5 keV Si PKAs and Ge PKAs are shown in Figure 15. Apparently, all the average lattice temperatures presented a descending trend from 0.1 ps to 100.5 ps, with two inflection points at about 0.2 ps and 35.5 ps. The average lattice temperatures stopped descending at 0.2 ps but resumed descending later at 1.5 ps, while at 35.5 ps, they stopped descending again and attempted at stabilizing over the remaining period of the collision cascade. The evolution of average lattice temperatures could thus be divided into three parts: the abrupt decline before 0.2 ps, the parabola-like decrease between 0.2 ps and 35.5 ps, and the stabilization after 35.5 ps, which generally aligned with three stages of the collision cascade, respectively. Moreover, with the PKA energy increasing from 1 keV to 5 keV, the average lattice temperatures became higher before 35.5 ps, especially those for the abrupt decrease before 0.2 ps, which indicates that PKAs with higher energy could transfer more energy to lattice atoms and induce a higher lattice temperature at the beginning of the collision cascade. After 35.5 ps, all average lattice temperatures were close and tended to stabilize at the ambient temperature of 300 K. Besides, for the same PKA energy, the average lattice temperatures of Si PKAs were quite close to those of Ge PKAs during the collision cascades. It signifies that different from the point defect, the average lattice temperature was mainly linked to the PKA energy and barely associated with the type and incidence direction of PKAs. In spite of that, the local lattice temperature could be influenced by the energy, type, and incidence direction of PKAs.

**Figure 16:**
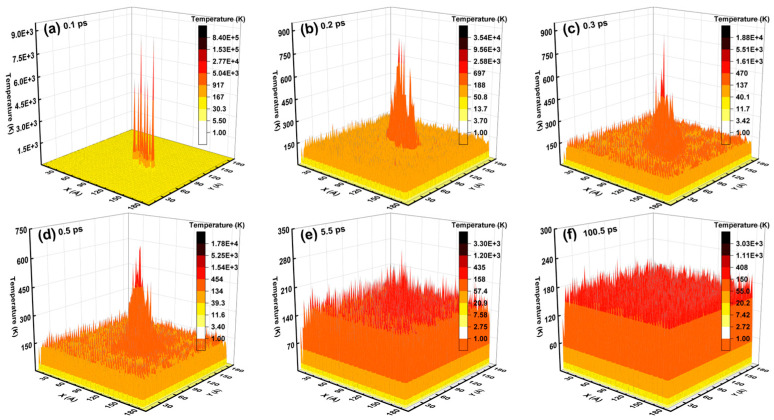
The spatial distribution of the lattice temperature inside the Newtonian region during an independent collision cascade induced by a 3 keV Ge PKA.

**Figure 17:**
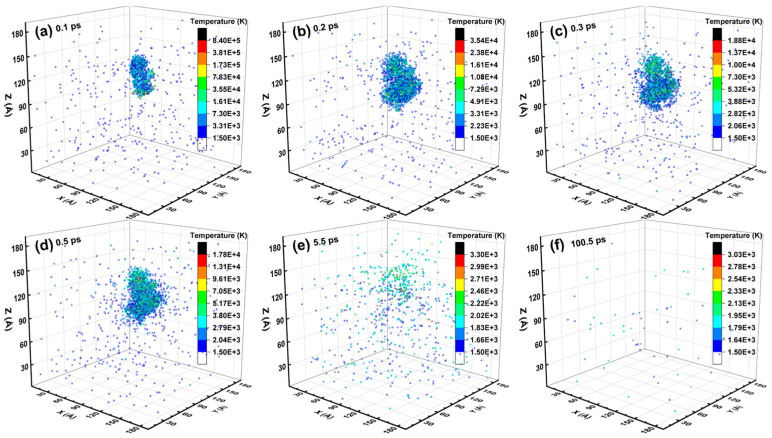
The spatial distribution of the melting region inside the Newtonian region during an independent collision cascade induced by a 3 keV Ge PKA.

Given the defect analysis above, the spatial distribution of the lattice temperature and the melting region inside the Newtonian region during an independent collision cascade induced by a 3 keV Ge PKA are displayed in Figure 16 and Figure 17, respectively. As regards the spatial distribution of the lattice temperature, the heat peaks at the center of the xy-plane declined throughout the collision cascade, with the heating area expanding from the center to the periphery of the Newtonian region. In detail, the heat peaks literally spiked at 0.1 ps, then remained in a similar shape from 0.2 ps to 0.5 ps, and almost disappeared at 5.5 ps. Meanwhile, the melting region was composed of a central melting lump and surrounding scattered areas. The melting lump first swelled abruptly from 0.1 ps to 0.2 ps, then basically kept the shape and size from 0.2 ps to 0.5 ps, and finally vanished at 5.5 ps, while the scattered areas faded away from 5.5 ps to 100.5 ps. During the whole period from 0.1 ps to 100.5 ps, the highest temperature of the melting lump was equal to that of the heat peaks, demonstrating that the melting lump dominated the spatial distribution of the lattice temperature. Besides, the nearly unchanged profile of the melting lump from 0.2 ps to 0.5 ps could be linked to the steady average lattice temperatures (in Figure 15).

**Figure 18:**
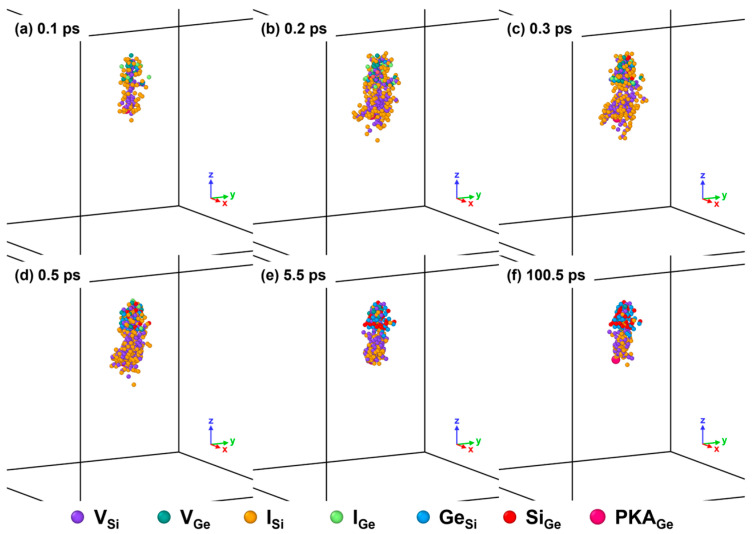
The spatial distribution of point defects inside the Newtonian region during an independent collision cascade induced by a 3 keV Ge PKA (the symbols of colored balls are available for the spatial distribution of point defects in other figures; the partial views of the spatial distribution are magnified on the same scale, which is applicable to similar subfigures in other figures).

Furthermore, the lattice atoms in the melting region were provided with higher energy than others, which empowered them to vibrate and migrate adequately, therefore contributing to the defect production. As presented in Figure 18, the outlines of the collection of six types of point defects at 0.1 ps, 0.2 ps, 0.3 ps, and 0.5 ps could match with those of the corresponding melting lump in the melting region, respectively. With the melting lump evolving, a collection of point defects expanded from 0.1 ps to 0.2 ps and remained in a similar shape and size from 0.2 ps to 0.5 ps. As the central melting lump disappeared at 5.5 ps, the point defects at the periphery of the defect collection might annihilate in a degree, but those in the center could still inherit the outline of the defect collection. Notably, the antisites in the SiGe alloy could survive in the defect collection after the collision cascade, which was consistent with the results of final point defects for 3 keV Ge PKAs.

**Figure 19:**
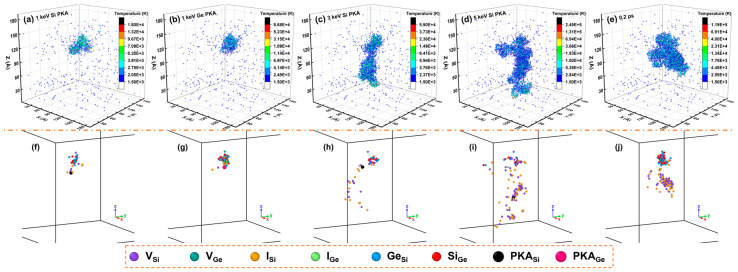
The spatial distribution of the melting region at 0.2 ps and the point defects at 100.5 ps inside the Newtonian region during independent collision cascades induced by a 1 keV Si PKA (**a**,**f**), 1 keV Ge PKA (**b**,**g**), 3 keV Si PKA (**c**,**h**), 5 keV Si PKA (**d**,**i**), and 5 keV Ge PKA (**e**,**j**).

Also, the spatial distribution of the melting region at 0.2 ps and the point defects at 100.5 ps inside the Newtonian region during independent collision cascades induced by a 1 keV Si PKA, 1 keV Ge PKA, 3 keV Si PKA, 5 keV Si PKA, and 5 keV Ge PKA, which had the same incidence direction as that of a 3 keV Ge PKA discussed above, are presented in Figure 19. Combined with the results for a 3 keV Ge PKA, for the same PKA type, the melting lump at 0.2 ps could be more prominent with the PKA energy increasing from 1 keV to 5 keV; for the same PKA energy, in comparison to a Si PKA, a Ge PKA could induce a compact melting lump at 0.2 ps. For 1 keV PKAs, the amorphous pocket was somewhat spherical, while for 5 keV PKAs, it could attempt to split. Likewise, as compared to a Si PKA of the same energy, a Ge PKA could retain more point defects at the end of the collision cascade, especially antisites, which was consistent with the results in the whole heterostructure during independent collision cascades. In this sense, the profile of a melting lump could be related to the discrepancy in the numbers of point defects between Si PKAs and Ge PKAs. A compact melting lump could bring about agglomerated point defects, where the point defects were difficult to escape and annihilate throughout the collision cascade. On the contrary, a large melting lump was inclined to split and release the point defects, which could be isolated and prone to annihilate during the collision cascade. Furthermore, in comparison to those for Si PKAs, the antisites in the SiGe alloy for Ge PKAs were inclined to agglomerate, which could enhance the final number of antisites for Ge PKAs.

Altogether, these results demonstrate that the melting lump gave birth to considerable point defects in the whole heterostructure. As compared to a Si PKA, a Ge PKA could induce a compact melting lump and bring about agglomerated point defects, which could easily survive in large part at the end of the collision cascade. Also, the final antisites for Ge PKAs are mainly sourced from the agglomeration of antisites in the SiGe alloy.

**Figure 20:**
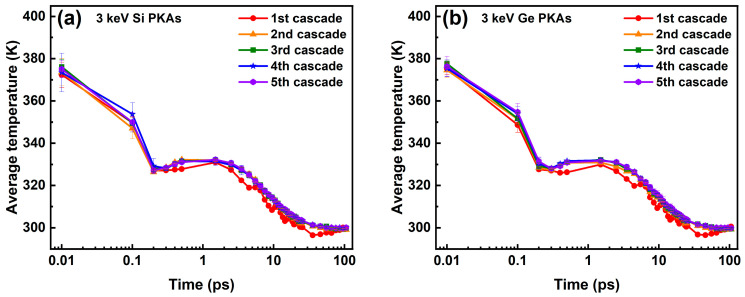
The average lattice temperatures of the Newtonian region in the SiGe/Si heterostructure during the overlapping cascades induced by (**a**) 3 keV Si PKAs and (**b**) 3 keV Ge PKAs.

Moreover, the average lattice temperatures of the Newtonian region during the overlapping cascades induced by 3 keV Si PKAs and Ge PKAs are shown in Figure 20. The average lattice temperatures of all five cascades for 3 keV Si PKAs and Ge PKAs evolved the same way as those of independent collision cascades induced by 3 keV Si PKAs and Ge PKAs, respectively. The average lattice temperatures of five cascades for 3 keV Si PKAs and Ge PKAs were close between cascades, respectively, although the average lattice temperature of the first cascade was slightly lower than those of the subsequent cascades during the period from 0.2 ps to 75.5 ps or so. The minor difference could be related to the damaged structure in the first cascade that altered the thermal conductivity of the SiGe/Si heterostructure. As reported by Scott et al. [74], after irradiated by several kinds of incident ions (including Si^2+^ and Ge^2+^), the thermal conductivity of silicon could be reduced, which was attributed to the structural disorder in silicon caused by the collision cascades. However, after the annealing, the thermal conductivity of the irradiated silicon could recover to a level comparable to that before irradiation. Herein, the approximate average lattice temperatures for the subsequent four cascades could be associated with the limited defects produced during the overlapping cascades, which did not much alter the thermal conductivity of the SiGe/Si heterostructure. Overall, these results suggest that the overlapping cascades had a minor effect on the evolution of average lattice temperatures in the SiGe/Si heterostructure.

**Figure 21:**
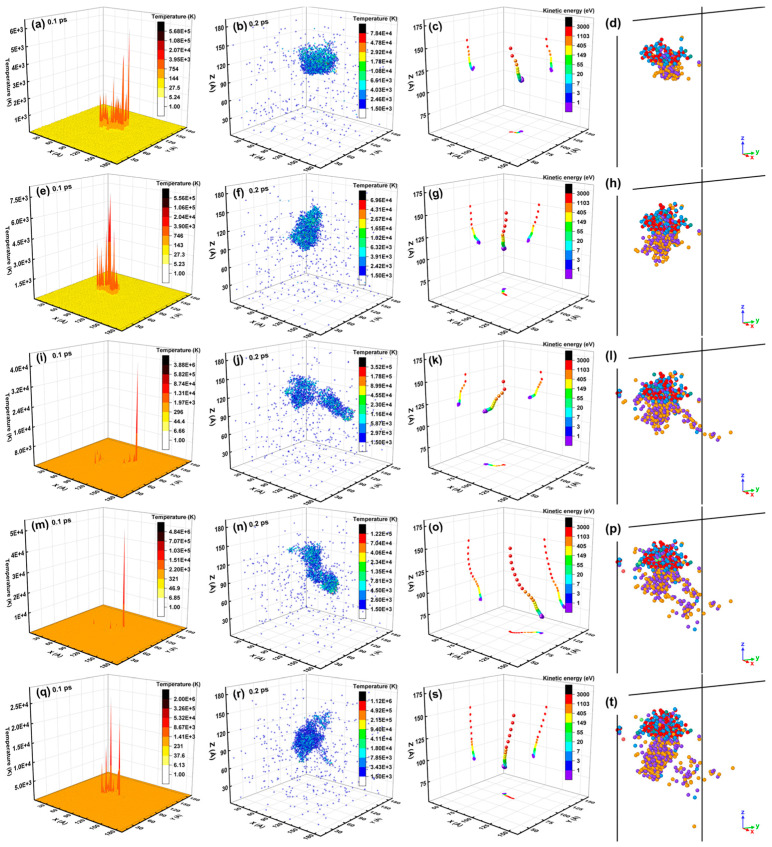
The spatial distribution of the lattice temperature at 0.1 ps, the melting region at 0.2 ps, the PKA track, and the point defect at 100.5 ps for a representative run of the overlapping cascades induced by 3 keV Ge PKAs: (**a**–**d**) the first cascade; (**e**–**h**) the second cascade; (**i**–**l**) the third cascade; (**m**–**p**) the fourth cascade; (**q**–**t**) the fifth cascade.

The spatial distribution of the lattice temperature at 0.1 ps, the melting region at 0.2 ps, the PKA track, and the point defects at 100.5 ps for a run of the overlapping cascades induced by 3 keV Ge PKAs are presented in Figure 21. The corresponding full views of the spatial distribution of point defects are also provided in Figure S10. Clearly, the outlines of the heat peaks and the melting lump were quite different among five cascades, and they could be apparently linked to the PKA tracks. In other words, different incidence directions of five PKAs led to different PKA tracks, which brought about different patterns of the energy dissipation of PKAs and the spatial distribution of the lattice temperature in the SiGe/Si heterostructure. Furthermore, during each cascade, the spatial distribution of surviving point defects could be controlled by the melting lump, especially for the first cascade. With the cascade number increasing, the outlines of the spatial distribution of point defects could be superimposed on each other and become larger, which was consistent with the evolution of final defects in the whole heterostructure. All in all, these results validate that during the overlapping cascades, the evolution of the lattice temperature was seldom influenced in each cascade, while the point defects could accumulate as the cascade number increased. The counterparts for a run of the overlapping cascades induced by 3 keV Si PKAs are also enclosed in Figure S11. As compared to 3 keV Ge PKAs, 3 keV Si PKAs could produce large melting lumps at 0.2 ps and result in few antisites in the SiGe alloy after the collision cascades.

In summary, the independent collision cascades induced by 1 keV, 3 keV, and 5 keV Si PKAs and Ge PKAs verify that the numbers of final Frenkel pairs and clusters in the whole heterostructure could be proportional to the total PKA energy, while those at the heterointerface were dominated by the PKA energy dissipated specifically nearby the heterointerface. Particularly, among all independent collision cascades, 3 keV Ge PKAs produced the most final antisites and the largest final clusters in the whole heterostructure and at the heterointerface. In virtue of their PKA tracks, 3 keV Ge PKAs could dissipate much energy around the heterointerface and generate a compact melting region in the SiGe/Si heterostructure, which hindered the recombination of Frenkel pairs and contributed to the formation of final antisites and clusters. In addition, the overlapping collision cascades induced by 3 keV Si PKAs and Ge PKAs reveal that the numbers of final point defects and clusters in the whole heterostructure could accumulate continually throughout five cascades. However, the numbers of final Frenkel pairs and clusters at the heterointerface could stop increasing in the fourth or the fifth cascades, which further affected the recombination ratio of Frenkel pairs and the formation of final clusters. On one hand, the proportional increase in final point defects and clusters in the whole heterostructure was directly linked to completely dissipated PKA energy as well as the mildly damaged structure, manifested by relatively independent average lattice temperatures in each cascade. On the other hand, the fluctuation of final point defects and clusters at the heterointerface could be associated with the annihilation of Frenkel pairs owing to the overlapping damage.

## 4. Conclusions

The primary radiation damage of a strain-engineering-based SiGe/Si heterostructure was investigated by classical MD simulations in two scenarios of independent and overlapping collision cascades. The main findings are as follows:

(1) Among 1 keV, 3 keV, and 5 keV Si PKAs and Ge PKAs, 5 keV Ge PKAs produced the most Frenkel pairs and clusters in the whole heterostructure, while 3 keV Ge PKAs acquired the most point defects (especially antisites) and clusters at the heterointerface.
(2) The numbers of point defects and clusters in the whole heterostructure induced by 3 keV Si PKAs and Ge PKAs could accumulate proportionally from the first to the fifth cascades, while those at the heterointerface tended to fluctuate in the fourth or the fifth cascades.
(3) The spatial distribution of surviving point defects in the whole heterostructure induced by 3 keV Si PKAs and Ge PKAs was dominated by the melting region, while it could be superimposed on the subsequent ones during the overlapping cascades.

The primary radiation damage in a SiGe/Si heterostructure can be correlated with many elements, such as the heterostructure model, cascade number, ambient temperature, and PKA conditions (energy, position, etc.), which cannot be fully covered in this work. Despite this, this work shall lay the foundations for understanding radiation effects of SiGe/Si heterostructures in SiGe-based electronics and developing radiation-hardening techniques.

## Data Availability

The original contributions presented in this study are included in the article/Supplementary Materials. Further inquiries can be directed to the corresponding authors.

## Supplementary Materials

The following supporting information can be downloaded at https://www.mdpi.com/article/10.3390/nano16030193/s1. Figure S1: A representative of the total energy evolution (a–e) and the final total energy (f) of the SiGe/Si heterostructure during each relaxation procedure of the overlapping cascades induced by 3 keV Si PKAs; Figure S2: A representative of the temperature (a–e) and the final temperature (f) of the SiGe/Si heterostructure during each relaxation procedure of the overlapping cascades induced by 3 keV Si PKAs; Figure S3: A representative of the stress (a–e) and the final stress (f) of the SiGe/Si heterostructure during each relaxation procedure of the overlapping cascades induced by 3 keV Si PKAs; Figure S4: A comparison of the PKA directions on the xy-plane for (a) 10 runs and (b) 20 runs of independent collision cascades induced by 5 keV Ge PKAs; Figure S5: A comparison of the numbers of six types of point defects in the whole heterostructure during the overlapping cascades induced by 5 keV Ge PKAs, corresponding to the PKA directions for 10 runs and 20 runs of simulations in Figure S4; Figure S6: The melting points of pure Si and the Si_0.7_Ge_0.3_ alloy obtained in MD simulations by the solid–liquid coexistence approach; Figure S7: The snapshots of atomic displacements during an independent collision cascade induced by a 3 keV Ge PKA: (a) 0 ps; (b) 0.01 ps; (c) 0.02 ps; (d) 0.03 ps; (e) 0.04 ps; (f) 0.05 ps; (g) 0.06 ps; (h) 0.08 ps; (i) 0.20 ps (each atom is colored by its kinetic energy, except the white balls that stand for the heterointerface); Figure S8: The six types of point defects in the whole heterostructure during the overlapping cascades induced by 3 keV Ge PKAs; Figure S9: The six types of point defects at the heterointerface during the overlapping cascades induced by 3 keV Ge PKAs; Figure S10: The full view of the spatial distribution of point defects for the corresponding subfigures in Figure 21: (a) the first cascade; (b) the second cascade; (c) the third cascade; (d) the fourth cascade; (e) the fifth cascade; Figure S11: The spatial distribution of the lattice temperature at 0.1 ps, the melting region at 0.2 ps, the PKA track, and the point defect at 100.5 ps for a representative run of the overlapping cascades induced by 3 keV Si PKAs: (a–d) the first cascade; (e–h) the second cascade; (i–l) the third cascade; (m–p) the fourth cascade; (q–t) the fifth cascade (the color of each cluster is randomly assigned to denote different clusters); Table S1: The electronic and nuclear energy loss of PKAs in pure Si and the Si_0.7_Ge_0.3_ alloy. Refs [65,66,75] are cited in the supplementary materials.
