# Supplementary material for: Primary Radiation Damage in a Strain-Engineering-Based SiGe/Si Heterostructure: A Molecular Dynamics Simulation
Source: Nanomaterials (Basel). 2026 Jan 30;16(3):193. doi: 10.3390/nano16030193 (PMC12899722; doi:10.3390/nano16030193)
Supplement: Supplementary file 1 [file nanomaterials-16-00193-s001.zip › nanomaterials-4112720-supplementary.pdf]

Supplementary Information for

Primary radiation damage in a strain-engineering-based SiGe/Si heterostructure: A molecular dynamics simulation

Tian Xing [a], Shuhuan Liu [a,*], Qian Wang [b], Chao Wang [a], Yuchen Wang [a], Mathew Adefusika Adekoya [a], Xuan Wang [a,*], Xinkun Li [a], Huawei Sheng [a], Luyang Cai [a], Jiatong Tan [a], Yalei Yi [a], Zhongliang Li [c,*]

[a] *School of Nuclear Science and Technology, Xi'an Jiaotong University, Xi'an 710049, China*

[b] *National Key Laboratory for Metrology and Calibration Techniques, Beijing 102413, China*

[c] *Shaanxi Qinzhou Nuclear and Radiation Safety Technology Co., Ltd., Xi'an 710054, China*

* Corresponding author: liushuhuan@mail.xjtu.edu.cn; wxuan@xjtu.edu.cn; lizhongliang@qznrs.net

Total number of pages: 12 excluding a cover page

Total number of tables: 1

Total number of figures: 11

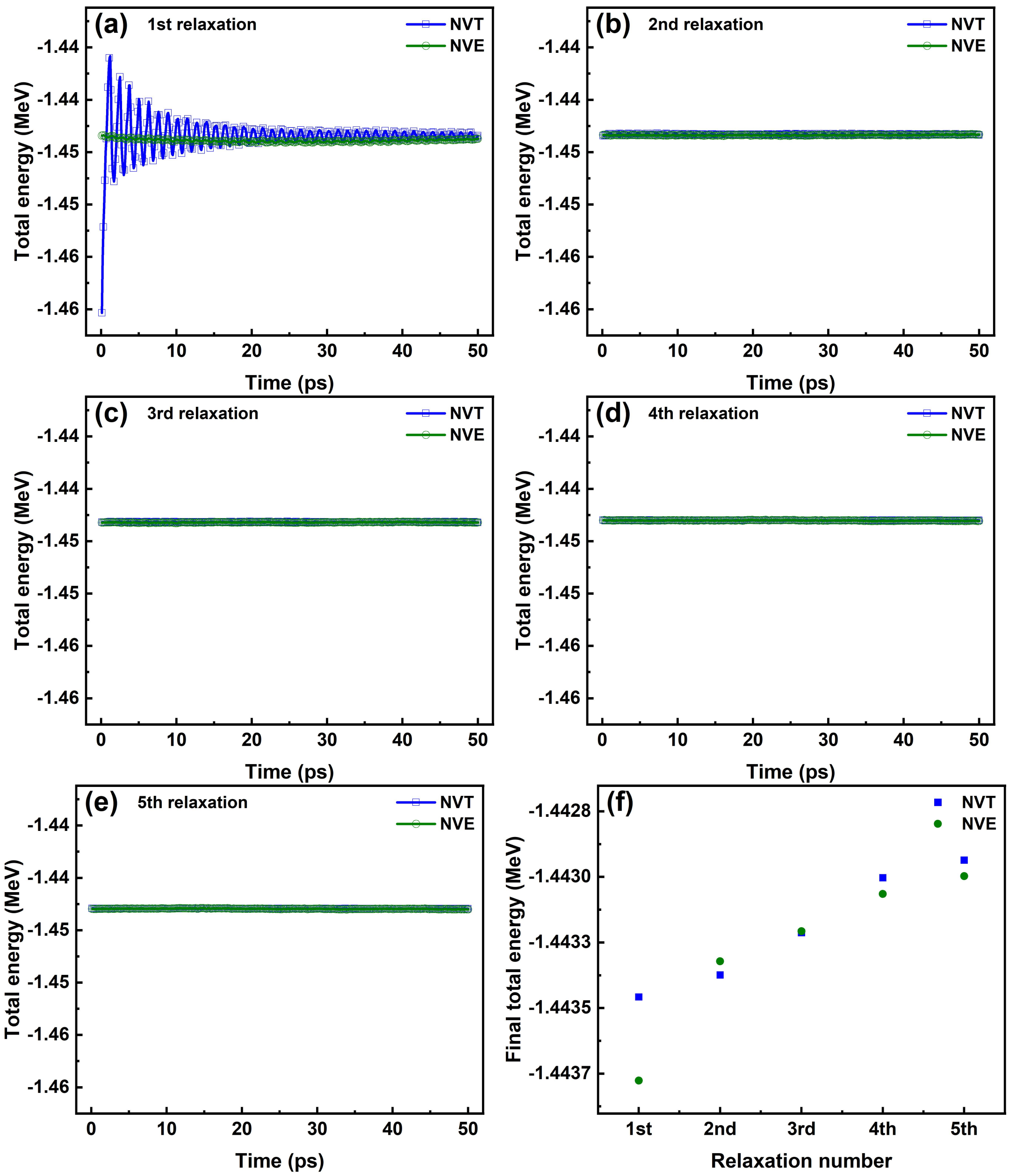

Figure S1. A representative of the total energy evolution (a–e) and the final total energy (f) of the SiGe/Si heterostructure during each relaxation procedure of the overlapping cascades induced by 3 keV Si PKAs.

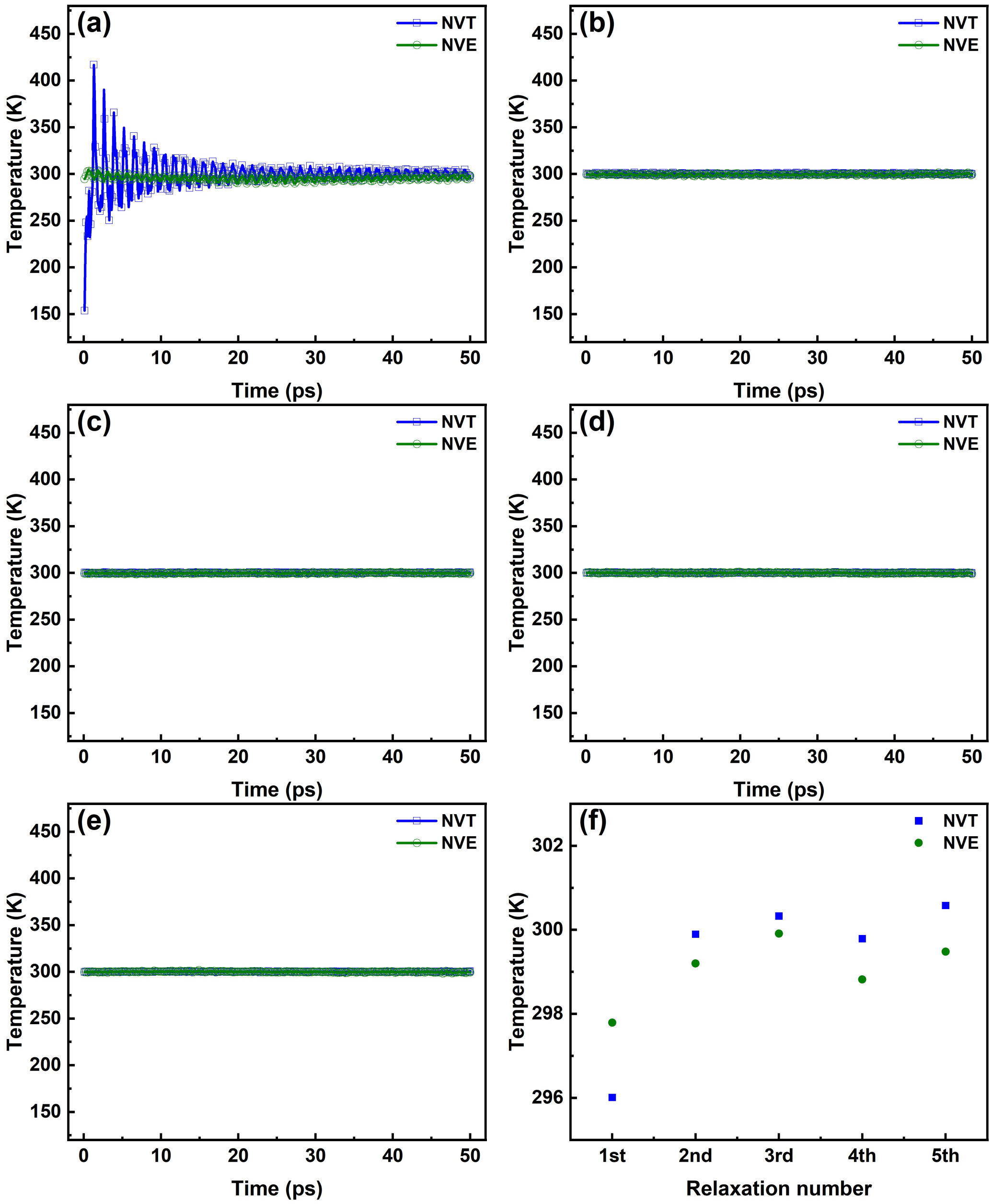

Figure S2. A representative of the temperature (a–e) and the final temperature (f) of the SiGe/Si heterostructure during each relaxation procedure of the overlapping cascades induced by 3 keV Si PKAs.

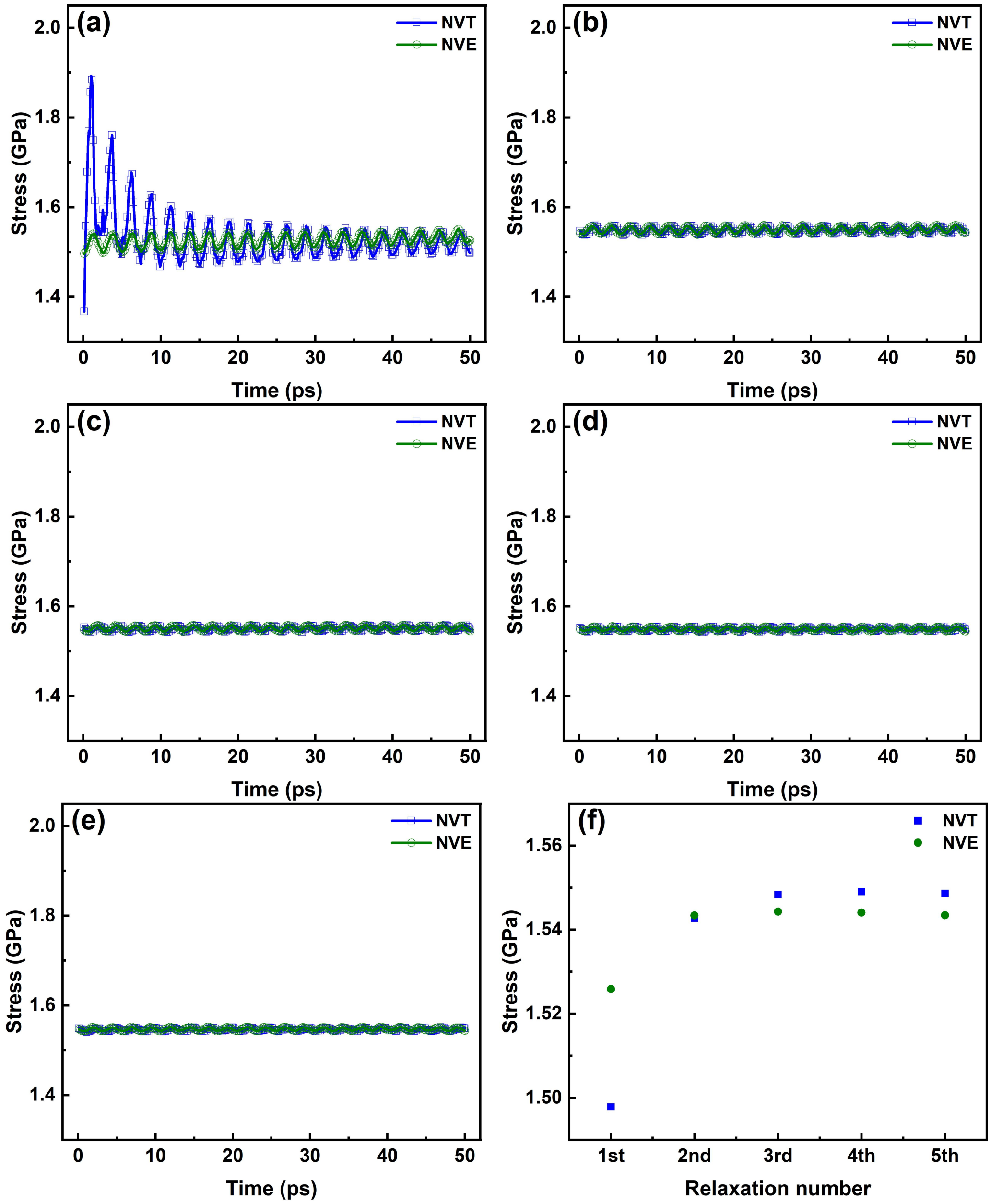

Figure S3. A representative of the stress (a–e) and the final stress (f) of the SiGe/Si heterostructure during each relaxation procedure of the overlapping cascades induced by 3 keV Si PKAs.

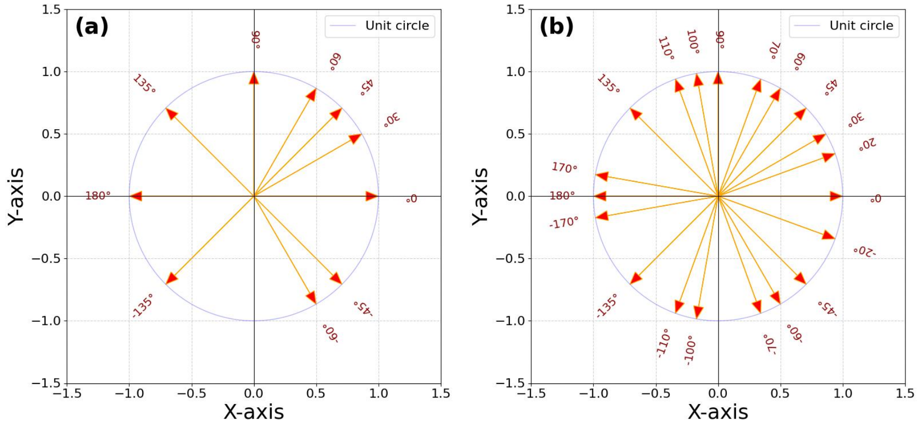

Figure S4. A comparison of the PKA directions on the xy-plane for (a) 10 runs and (b) 20 runs of independent collision cascades induced by 5 keV Ge PKAs.

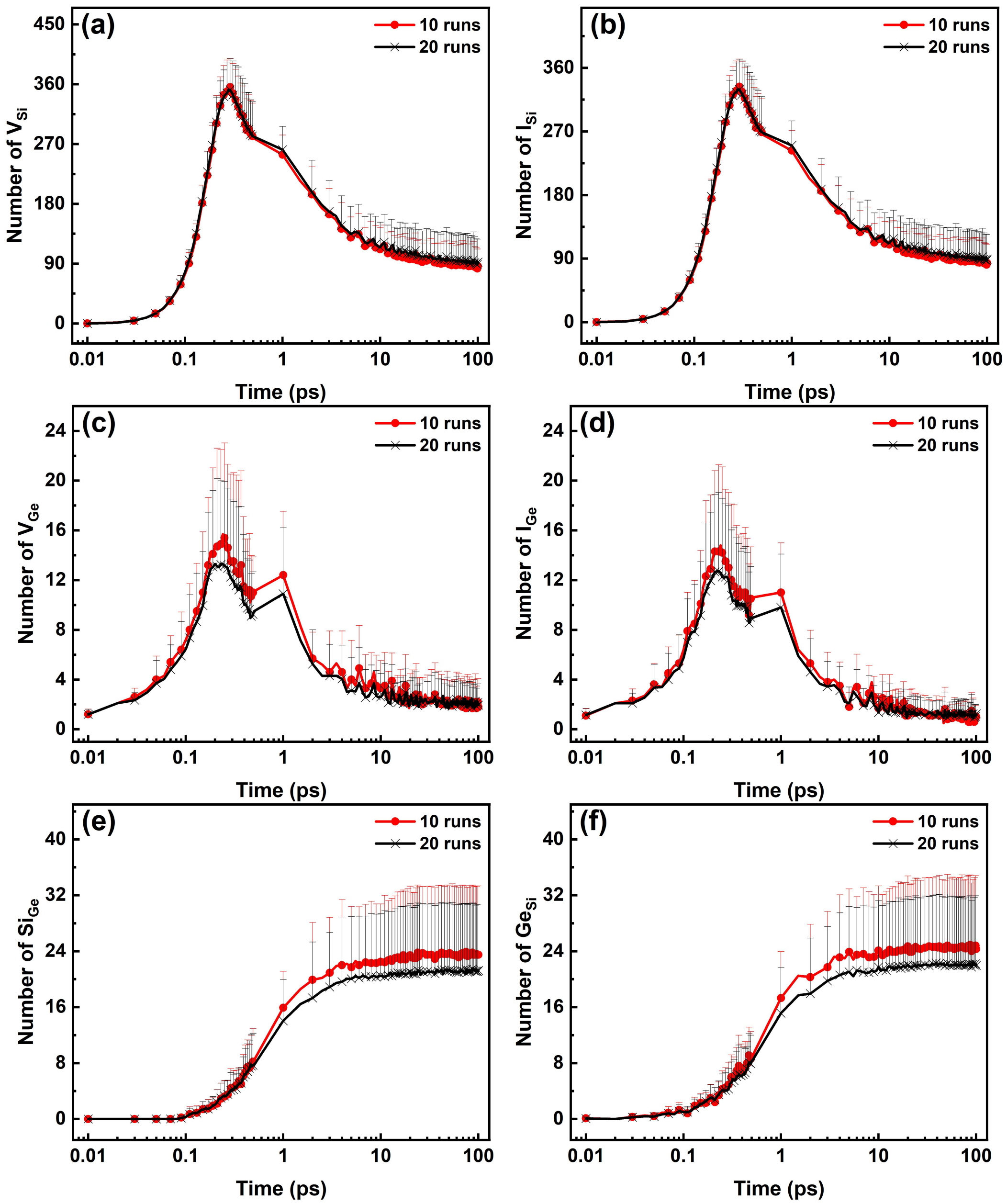

Figure S5. A comparison of the numbers of six types of point defects in the whole heterostructure during the overlapping cascades induced by 5 keV Ge PKAs, corresponding to the PKA directions for 10 runs and 20 runs of simulations in **Figure S4**.

Table S1. The electronic and nuclear energy loss of PKAs in pure Si and the $Si_{0.7}Ge_{0.3}$ alloy.

PKA in target	PKA energy	S_e (MeV/(mg/cm²))	S_n (MeV/(mg/cm²))	S_e/S_n	$S_e/(S_e + S_n)$
Si PKA in Si	1 keV	0.15	1.06	13.7%	12.0%
	3 keV	0.25	1.43	17.6%	15.0%
	5 keV	0.32	1.57	20.7%	17.2%
Ge PKA in Si	1 keV	0.07	1.77	4.2%	4.0%
	3 keV	0.13	2.76	4.7%	4.5%
	5 keV	0.17	3.28	5.1%	4.8%
Si PKA in $Si_{0.7}Ge_{0.3}$	1 keV	0.10	0.71	14.4%	12.6%
	3 keV	0.18	0.97	18.2%	15.4%
	5 keV	0.23	1.07	21.2%	17.5%
Ge PKA in $Si_{0.7}Ge_{0.3}$	1 keV	0.05	1.29	4.0%	3.9%
	3 keV	0.09	2.03	4.5%	4.3%
	5 keV	0.12	2.43	4.8%	4.6%

The calculation method of the number of Frenkel pairs

According to the NRT model [1,2], the number of surviving Frenkel pairs can be calculated as follows:

$$N_d = 0.8T_d/2E_d^{eff} \tag{S1}$$

where T_d was the damage energy of PKAs (the PKA energy minus the electronic stopping energy), and E_d^{eff} was the effective threshold displacement energy (TDE) and calculated as follows [2]:

$$E_d^{eff} = \left[\sum_i \frac{S_i}{E_{d,i}}\right]^{-1} \tag{S2}$$

where S_i and $E_{d,i}$ were the stoichiometric ratio and the TDE of the i element in a multielement compound, respectively. Accordingly, the effective TDE of the SiGe/Si heterostructure was calculated to be 32.1 eV based on the TDE of Si (33 eV) and Ge (23 eV) [2,3].

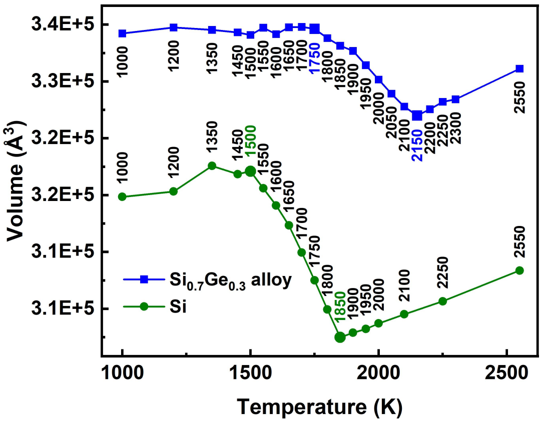

Figure S6. The melting points of pure Si and the $Si_{0.7}Ge_{0.3}$ alloy obtained in MD simulations by the solid–liquid coexistence approach.

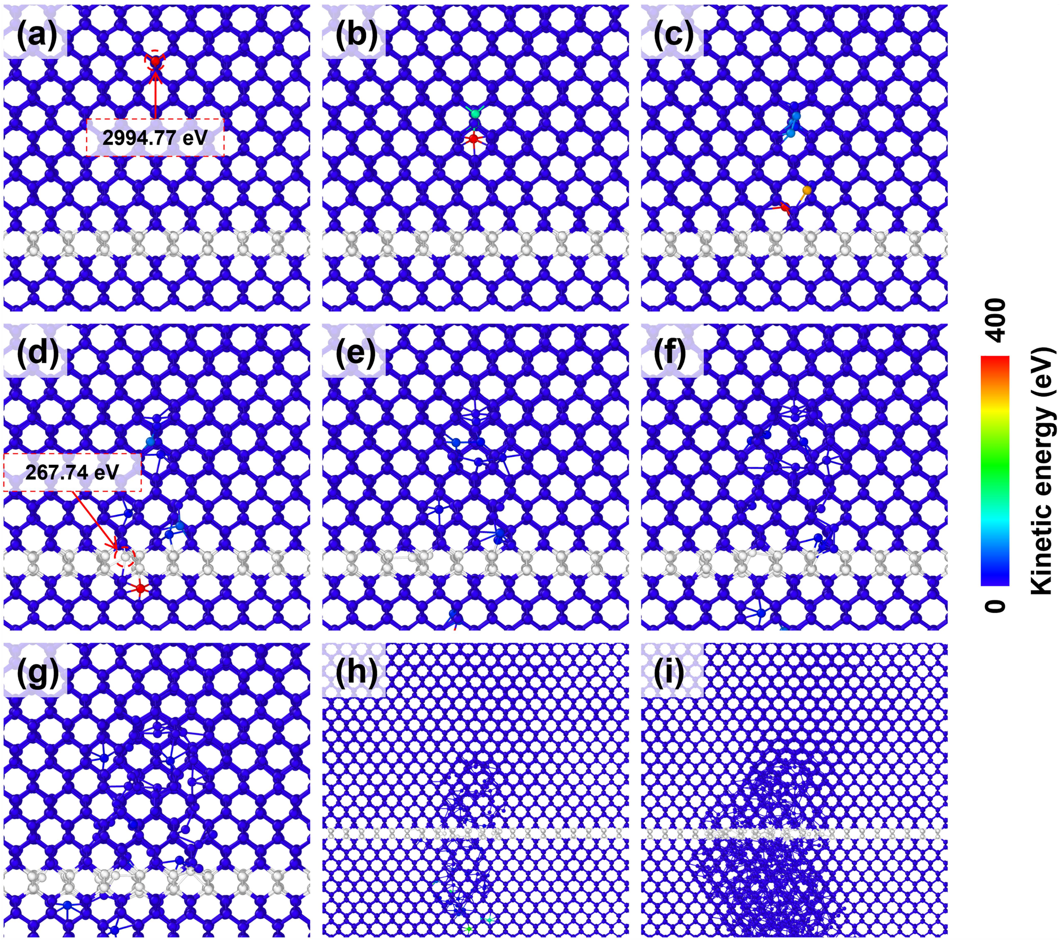

Figure S7. The snapshots of atomic displacements during an independent collision cascade induced by a 3 keV Ge PKA: (a) 0 ps; (b) 0.01 ps; (c) 0.02 ps; (d) 0.03 ps; (e) 0.04 ps; (f) 0.05 ps; (g) 0.06 ps; (h) 0.08 ps; (i) 0.20 ps (each atom is colored by its kinetic energy, except the white balls that stand for the heterointerface).

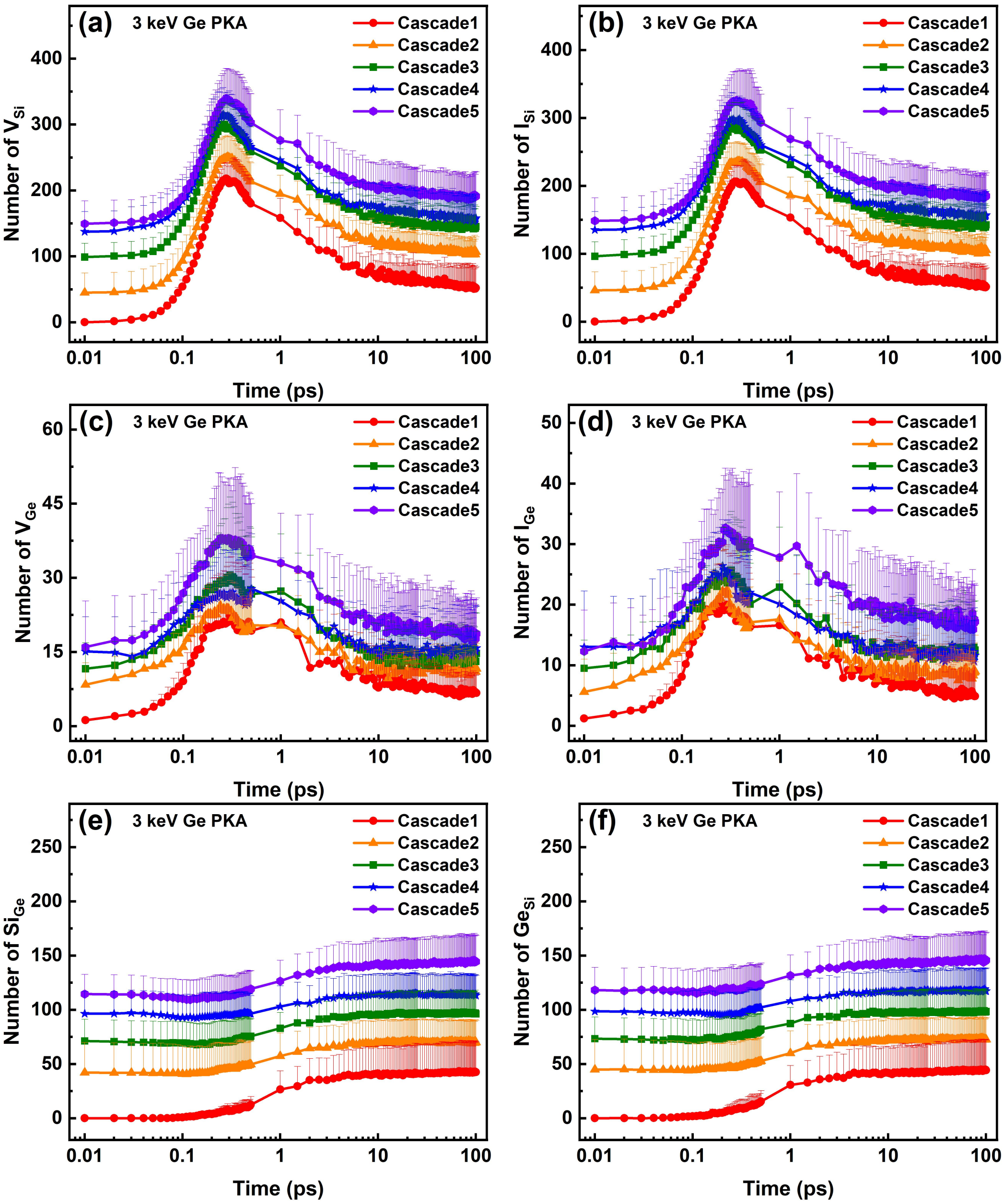

Figure S8. The six types of point defects in the whole heterostructure during the overlapping cascades induced by 3 keV Ge PKAs.

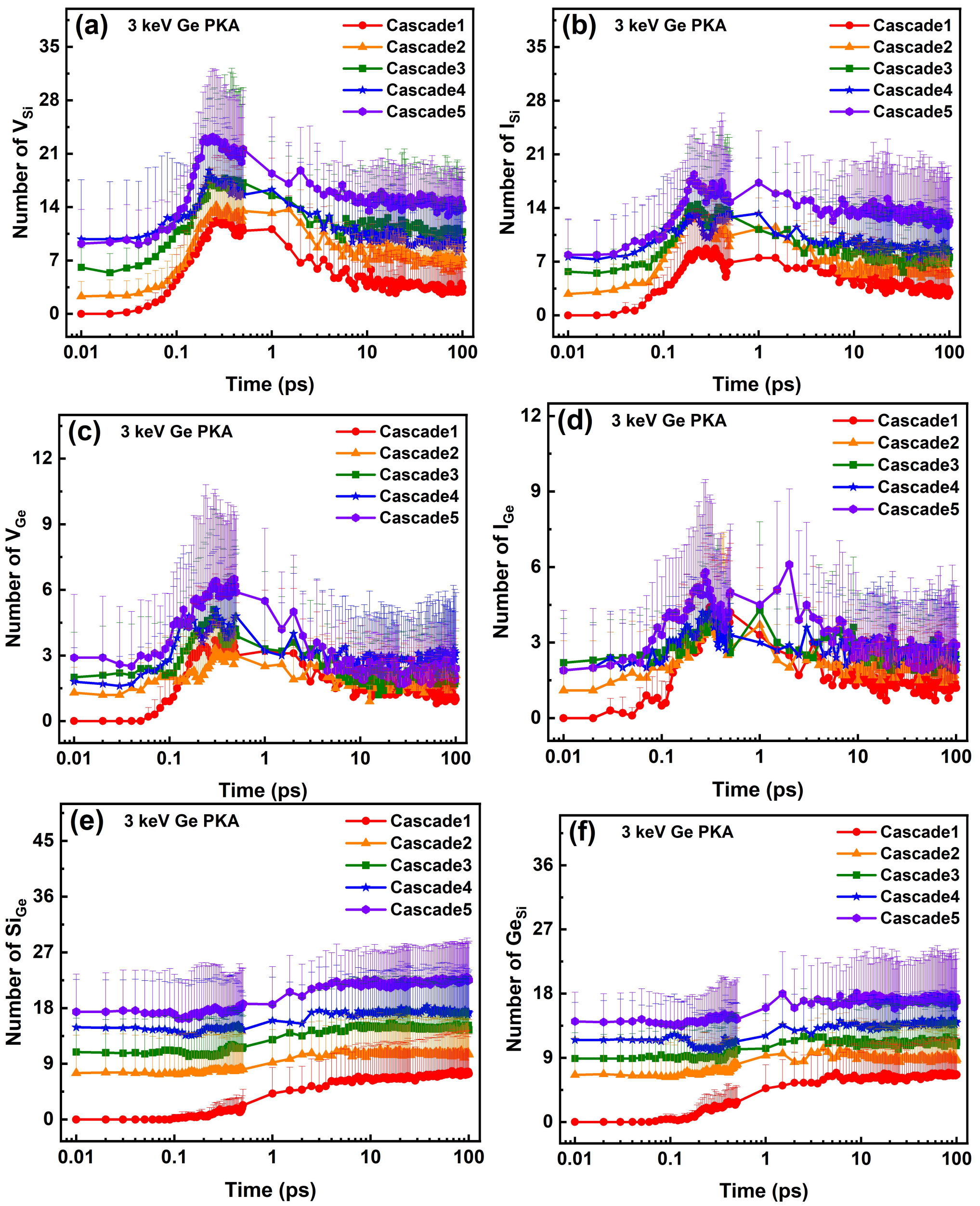

Figure S9. The six types of point defects at the heterointerface during the overlapping cascades induced by 3 keV Ge PKAs.

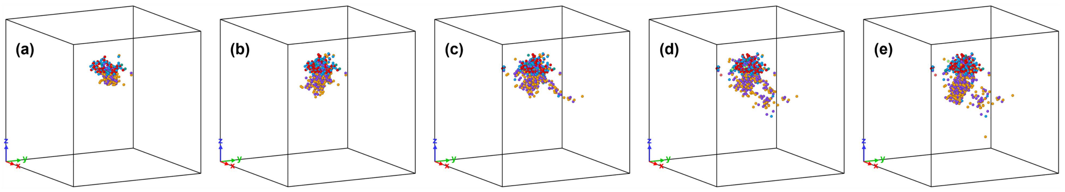

Figure S10. The full view of the spatial distribution of point defects for the corresponding subfigures in Figure 21: (a) the first cascade; (b) the second cascade; (c) the third cascade; (d) the fourth cascade; (e) the fifth cascade.

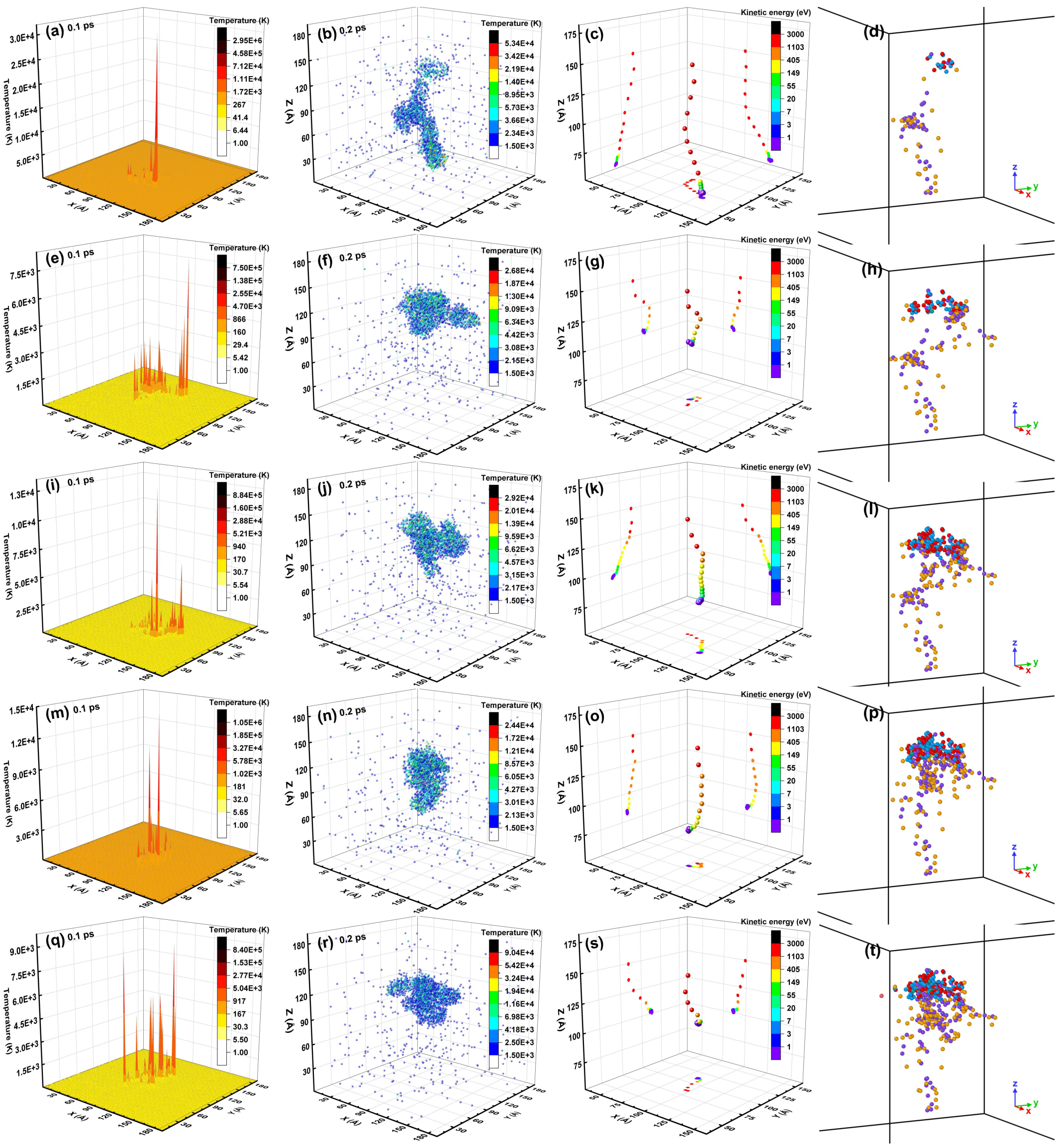

Figure S11. The spatial distribution of the lattice temperature at 0.1 ps, the melting region at 0.2 ps, the PKA track, and the point defect at 100.5 ps for a representative run of the overlapping cascades induced by 3 keV Si PKAs: (a–d) the first cascade; (e–h) the second cascade; (i–l) the third cascade; (m–p) the fourth cascade; (q–t) the fifth cascade.

References

1. Nordlund, K.; Zinkle, S.J.; Sand, A.E.; Granberg, F.; Averback, R.S.; Stoller, R.E.; Suzudo, T.; Malerba, L.; Banhart, F.; Weber, W.J.; et al. Primary radiation damage: A review of current understanding and models. *Journal of Nuclear Materials* **2018**, *512*, 450-479, doi:https://doi.org/10.1016/j.jnucmat.2018.10.027.
2. Agarwal, S.; Lin, Y.; Li, C.; Stoller, R.E.; Zinkle, S.J. On the use of SRIM for calculating vacancy production: Quick calculation and full-cascade options. *Nuclear Instruments and Methods in Physics Research Section B: Beam Interactions with Materials and Atoms* **2021**, *503*, 11-29, doi:https://doi.org/10.1016/j.nimb.2021.06.018.
3. Holmström, E.; Nordlund, K.; Kuronen, A. Threshold defect production in germanium determined by density functional theory molecular dynamics simulations. *Physica Scripta* **2010**, *81*, 035601, doi:https://doi.org/10.1088/0031-8949/81/03/035601.
